# Protein Nanoparticle-Mediated Delivery of Recombinant Influenza Hemagglutinin Enhances Immunogenicity and Breadth of the Antibody Response

**DOI:** 10.1021/acsinfecdis.2c00362

**Published:** 2023-01-06

**Authors:** Alexander J. Badten, Aaron Ramirez, Jenny E. Hernandez-Davies, Tyler J. Albin, Aarti Jain, Rie Nakajima, Jiin Felgner, D. Huw Davies, Szu-Wen Wang

**Affiliations:** ^†^Department of Chemical and Biomolecular Engineering, ^‡^Vaccine Research and Development Center, Department of Physiology and Biophysics, ^§^Department of Chemistry, ^∥^Department of Biomedical Engineering, ^⊥^Chao Family Comprehensive Cancer Center, ^#^Institute for Immunology, University of California, Irvine, California 92697, United States

**Keywords:** protein nanoparticle, influenza vaccine, maleimide tris-NTA, E2, homosubtypic cross-reactivity, heterosubtypic cross-reactivity, hemagglutinin

## Abstract

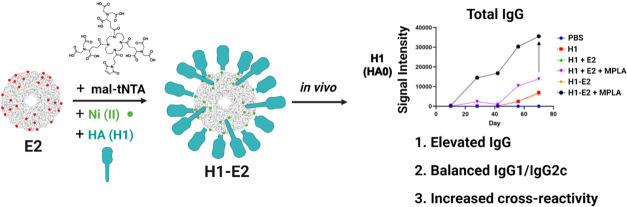

The vast majority of seasonal influenza vaccines administered each year are derived from virus propagated in eggs using technology that has changed little since the 1930s. The immunogenicity, durability, and breadth of response would likely benefit from a recombinant nanoparticle-based approach. Although the E2 protein nanoparticle (NP) platform has been previously shown to promote effective cell-mediated responses to peptide epitopes, it has not yet been reported to deliver whole protein antigens. In this study, we synthesized a novel maleimido tris-nitrilotriacetic acid (NTA) linker to couple protein hemagglutinin (HA) from H1N1 influenza virus to the E2 NP, and we evaluated the HA-specific antibody responses using protein microarrays. We found that recombinant H1 protein alone is immunogenic in mice but requires two boosts for IgG to be detected and is strongly IgG1 (Th2) polarized. When conjugated to E2 NPs, IgG2c is produced leading to a more balanced Th1/Th2 response. Inclusion of the Toll-like receptor 4 agonist monophosphoryl lipid A (MPLA) significantly enhances the immunogenicity of H1–E2 NPs while retaining the Th1/Th2 balance. Interestingly, broader homo- and heterosubtypic cross-reactivity is also observed for conjugated H1–E2 with MPLA, compared to unconjugated H1 with or without MPLA. These results highlight the potential of an NP-based delivery of HA for tuning the immunogenicity, breadth, and Th1/Th2 balance generated by recombinant HA-based vaccination. Furthermore, the modularity of this protein–protein conjugation strategy may have utility for future vaccine development against other human pathogens.

Recombinant protein vaccines are inherently safer than live attenuated vaccines since they pose no risk of reversion to a virulent phenotype and can be used in immunocompromised individuals. Recombinant proteins also obviate the need for propagation of the pathogen, which may introduce mutations (as is the case for influenza virus propagated in hen eggs^1−5^), or pose safety concerns if the pathogen needs to be grown at high containment (BSL3 or 4). It is also challenging to control amounts of antigen with live vaccines, which can give rise to toxicity concerns, immunodominance of nonprotective antigens, or immune subversion caused by immunomodulatory materials.^6,7^ However, recombinant proteins tend to have weaker immunogenicity than live attenuated vaccines, caused by factors such as rapid draining kinetics, monovalency of vaccine antigens, reduced capacity to stimulate innate immunity through pattern recognition receptors (PRRs), and differential pharmacokinetics of vaccine components.^8−10^ This generally requires such vaccines to be administered with immunoenhancing substances (collectively termed “adjuvants”) such as emulsions and pattern recognition receptor (PRR) agonists, and typically in multiple (booster) doses to achieve adequate immunity.^11^

NP-based vaccine delivery systems are a promising solution, combining the safety and tunability of subunit vaccines with the strong immunogenicity of particulate antigen.^12−15^ This phenomenon is primarily due to two unique properties of nanoparticles (NPs): their increased size relative to soluble antigen and the repetitive pattern in which antigens are displayed on their surface. Experimental and computational studies have indicated that dendritic cells preferentially take up nanoparticles smaller than 500 nm with an optimal uptake size of ca. 25–50 nm.^16−20^ Diameters larger than 25 nm also have increased retention times within draining lymph nodes.^16−20^ Previous studies of nanoparticle (NP) scaffolds with controlled antigen valencies have also suggested that the antibody-producing B cells of the adaptive immune system are more efficiently activated by five or more repeated epitopes, via improved B cell receptor (BCR) cross-linking and subsequent activation.^21−23^

NPs have received attention in tumor^24−29^ and autoimmune disease^30−33^ models due to their capacity to elicit strong cytotoxic T lymphocyte (CTL) and regulatory T cell responses (T-reg), respectively, to peptide epitopes. However, B cell epitopes often require specific three-dimensional conformations that are generally not represented by peptide fragments.^34−36^ Therefore, there is a need to attach full-length protein antigens onto NPs. One strategy to accomplish this is genetically fusing the antigen to a protein that naturally self-assembles into a virus-like particle (VLP).^37−41^ However, genetic fusion frequently leads to protein misfolding or expression issues.^42,43^ For this reason, alternative methods have been explored to attach full-length proteins to various NP platforms post-assembly, both covalently^44−46^ and noncovalently.^47,48^

In this work, we apply Ni(II)-chelated nitrilotriacetic acid (NTA), which has an affinity for polyhistidine-tagged proteins,^49−51^ as a method for attachment of influenza hemagglutinin (HA) to an NP assembled from the E2 subunit of pyruvate dehydrogenase (PDH) (see below). To overcome the relatively low binding affinity of Ni-NTA to hexahistidine (*K*_D_ of ∼13 μM^52,53^), we used a cyclic tris-NTA, which elicits 3–4 orders of magnitude higher affinities to His_6_ tags than monovalent Ni-NTA, with a *K*_D_ of ca. 2–20 nM.^53,54^ To accomplish this, a maleimide functional group was added to tris-NTA to conjugate to the cysteine residues on our E2 NP scaffold. Although the presence of a repetitive structural array of antigens and uniform antigen decoration is reported to enhance B-cell activation and antibody responses, there are currently limited options in the toolbox of short chemical linkers for attaching protein antigens to the surface of a nanoparticle while maintaining the same geometric orientation.^10,55^ Therefore, the synthesis and development of a tris-NTA linker could more broadly enable relatively straightforward, modular assembly of NP-based vaccines using any polyhistidine-tagged antigen.

E2 is a subunit of the *Geobacillus stearothermophilus* PDH complex that self-assembles into a 60-mer hollow spherical protein cage of ∼25 nm diameter^56,57^ and can be functionalized with non-native molecules on its external and internal surfaces.^58−60^ We have previously shown that this platform can efficiently activate dendritic cells^61^ and elicit CD8 T cell responses in tumor vaccination models when using CD8 epitope peptide antigens.^28,62,63^ Here, we predicted that attaching a protein antigen to our E2 nanoparticle using a novel tris-NTA linker would yield a favorable size (relative to soluble antigen) that allows for B cell receptor cross-linking^22,64^ and antibody production. To test this, we have conjugated an antigen protein to the surface of E2 for the first time, specifically the 523-amino acid influenza HA protein (subtype H1 from A/California/7/2009), and show that it engenders a quantitatively enhanced antibody response to H1 compared to H1 + E2 administered separately. We also show that administration of H1–E2 NPs in an adjuvant comprising toll-like receptor 4 (TLR4) agonist (monophosphoryl lipid A; MPLA) enhances the magnitude and breadth of the response over nonadjuvanted formulations.

## Results and Discussion

Seasonal influenza, caused by influenza A and B viruses, results in 290,000–650,000 deaths annually worldwide.^65^ In this study, we selected influenza A virus (IAV) as our pathogen model to develop an E2 nanoparticle (NP) vaccine, due to its relevance to human health, the detailed understanding of influenza vaccinology, and the availability of many influenza proteins with His-tags suitable for Ni-NTA conjugation chemistry.^66,67^ Attachment of virions to the host cell is mediated by binding of hemagglutinin (HA) present on the virion surface to cell-surface sialic acid resides. HA is also the immunodominant target of virus-neutralizing antibodies and a lead vaccine antigen. Of the 18 known IAV hemagglutinin subtypes (H1–H18), only H1 and H3 are currently found circulating in humans, and we have chosen to focus on H1 in these studies due to its importance in seasonal influenza in humans.

Structurally, HA is expressed in the viral membrane as a highly glycosylated homotrimer with each monomer consisting of a single polypeptide demarcated into two distinct regions by a cleavage site: HA1, which contains the highly variable head region and part of the more conserved stem region, and HA2, which encodes the remainder of the stem (Figure SI-1). Here we speculated that our E2 NP, which is of a size (∼25 nm) particularly suited to antigen-presenting cell (APC) uptake, and its ability to present multiple HA proteins in a regular, repetitive pattern, would lead to the elicitation of superior immunity compared to a free antigen control.

### Synthesis of Maleimido Cyclic tris-NTA

**Scheme 1:**
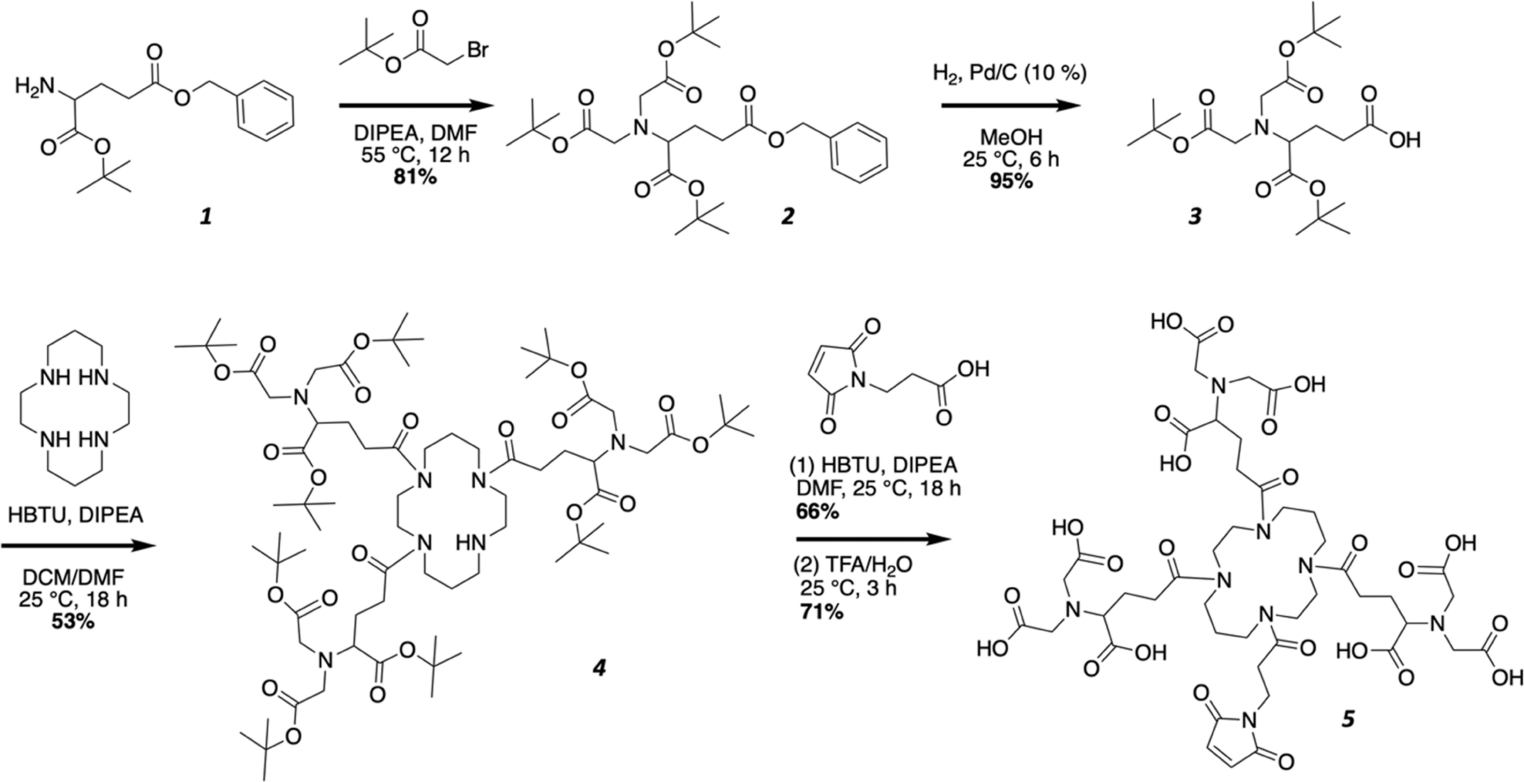
Synthesis Route of mal-tNTA **1** = H-l-Glu(Bzl)-OtBu*HCl, **2** = di-*t*-butylacetate-l-Glu(Bzl)-OtBu, **3** = di-*t*-butylacetate-l-Glu-OtBu, **4** = *t*-butyl-protected tris-NTA-NH, **5** = tris-NTA-mal (mal-tNTA).

To perform the conjugation of the protein antigen to the E2 NP, we generated a maleimido cyclic tris-NTA (mal-tNTA) as described in the Materials and Methods section. By the synthetic route shown in Scheme 1, hundreds of milligrams of mal-tNTA were readily produced, enabling antigen conjugation to the NPs. Liquid chromatography–mass spectrometry (LC–MS) was used to confirm the identity of mal-tNTA (Figure SI-2). Mal-tNTA is significant as a new linker for joining His-tagged proteins to thiol-functionalized materials, such as cysteine-containing proteins, enabling modular assembly of different protein NPs and antigens. Previous uses of tNTA on NPs involved the use of tNTA linkers with lipid tails that allowed for embedding into liposomes, but here we show a covalent method of attaching tNTA onto protein NPs via maleimide.^68−70^ The tris-NTA linker has previously been applied to delay the release of antigens and deliver genome-editing proteins from liposome NPs *in vitro*, though *in vivo* studies have not corroborated these findings.^68−70^ Nonetheless, *in vivo* studies utilizing tris-NTA to bind protein antigens to liposome NPs have shown elevated antibody responses compared to unbound protein antigen and efficacy in a tumor model.^68,71^

### Surface Display and Attachment of His-Tagged Influenza Hemagglutinin Protein to E2 Nanoparticle

#### Attachment of mal-tNTA to E2

**Figure 1:**
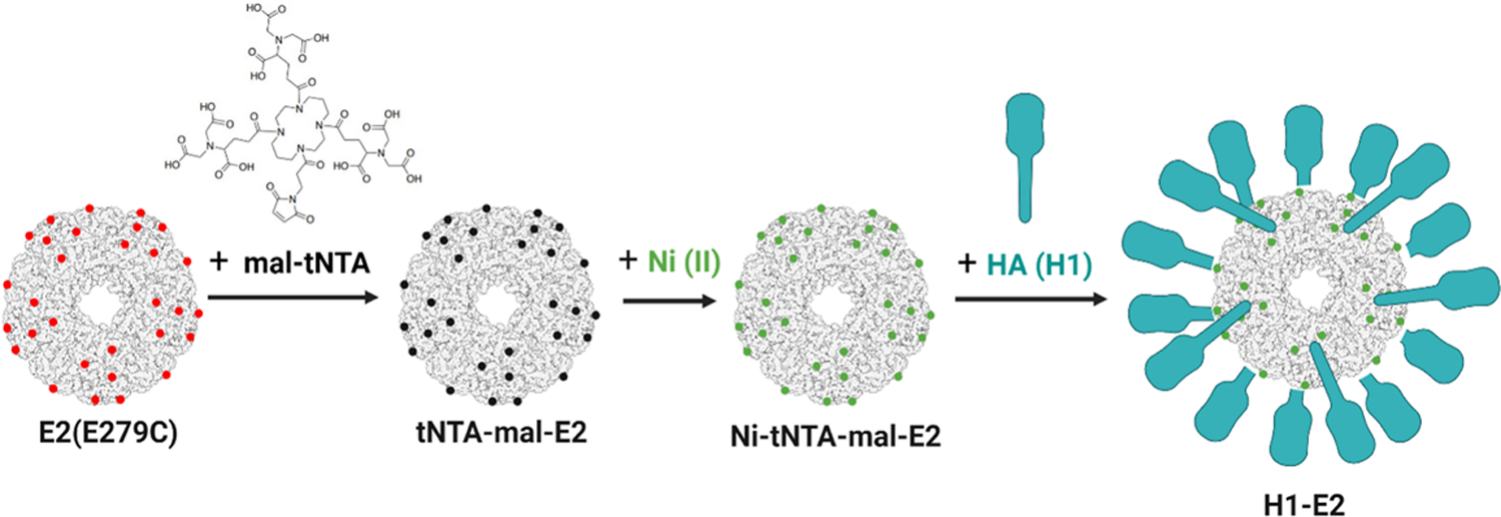
Hemagglutinin (subtype H1) protein attachment to E2 using mal-tNTA. The E2 mutant, E279C, is a 60-subunit protein nanoparticle assembly that contains 60 cysteines on the surface (red points). Maleimide-tNTA is conjugated to these external cysteines (black points), and Ni(II) is loaded onto tNTA via chelation (green points). A polyhistidine tag on the H1 antigen associates with the Ni-chelated tNTAs to form a coordination bond, resulting in a nanoparticle displaying H1 on the surface.

**Figure 2:**
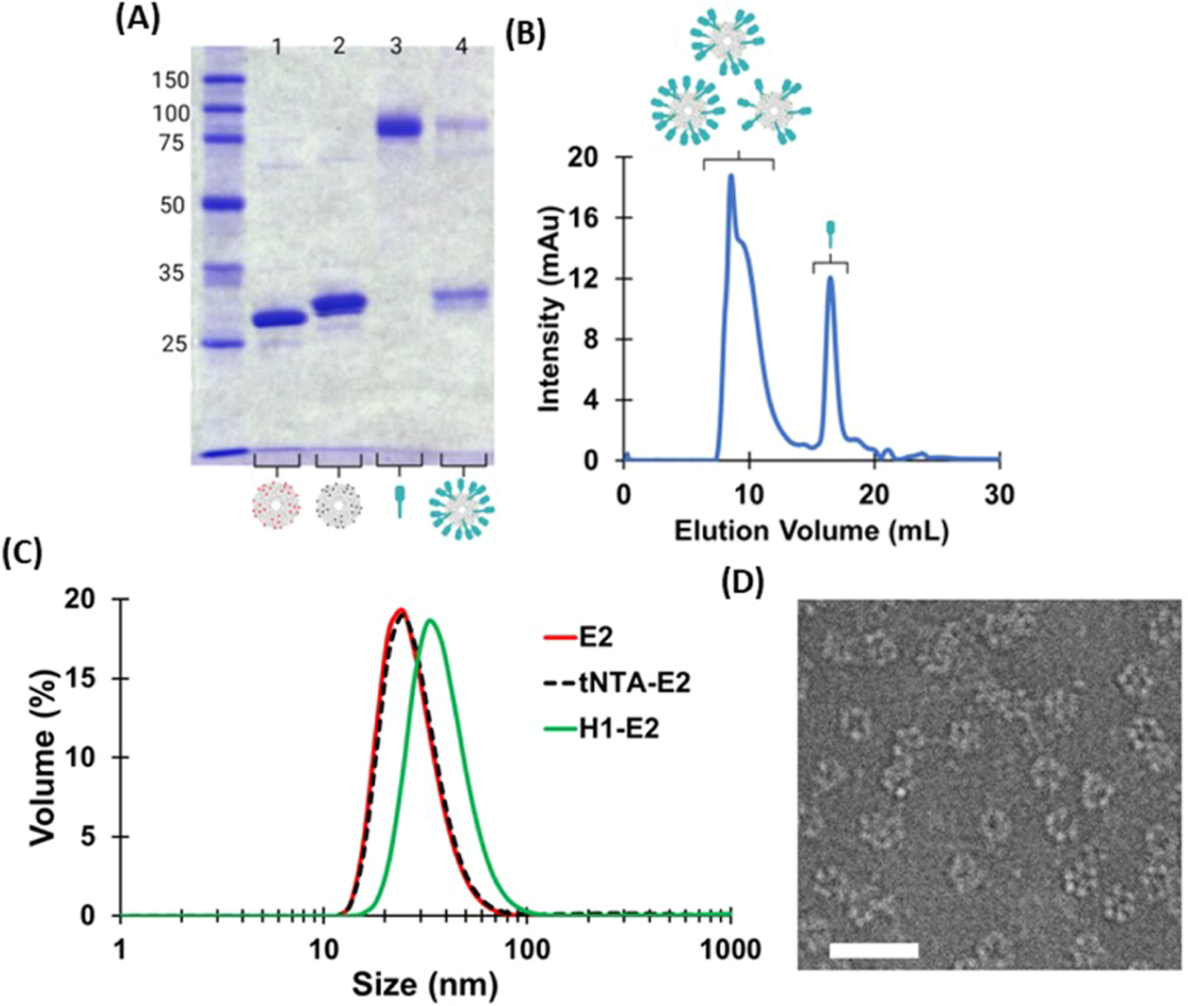
Characterization of tNTA-E2 and H1–E2 nanoparticles. (A) SDS-PAGE showing E2 (E279C) alone (lane 1), tNTA-E2 (lane 2), H1 alone (lane 3), and H1–E2 (lane 4). (B) Representative chromatogram of the size exclusion chromatography (SEC) performed to separate unreacted HA. Each collected fraction was analyzed with SDS-PAGE (Figure SI-4) and confirmed H1-bound E2 in the first SEC peak and unbound H1 in the second peak. (C) Hydrodynamic diameters for E2, tNTA-E2, and H1–E2, and average sizes were 27.3 ± 1.1, 28.8 ± 2.2, and 38.2 ± 1.7 nm, respectively. (D) Representative transmission electron microscopy (TEM) image of H1–E2 nanoparticles. Scale bar = 50 nm.

HA was successfully attached on the surface of the E2 NP as shown in Figures 1 and 2. The conjugation of mal-tNTA to E2 was supported by the ∼1 kDa band shift on sodium dodecyl-sulfate polyacrylamide gel electrophoresis (SDS-PAGE) (Figure 2A) and confirmed by mass spectrometry (Figure SI-3). The theoretical molecular weights of E2 (E279C mutant) and mal-tNTA conjugated to E2 are 28091 and 29179 Da, respectively. Experimentally, we obtained molecular weights of 28091 ± 0 Da for E2 (E279C; *n* = 3) and 29177 ± 0.5 Da for mal-tNTA-E2 (*n* = 3), both of which closely match predicted values. Both SDS-PAGE and MS data show that the conjugation yield was >90%. The resulting mal-tNTA-E2 nanoparticles also structurally remained intact, resulting in a single peak at an average hydrodynamic diameter of 28.8 ± 2.2 nm, which is similar to the size of E2-alone (27.3 ± 1.1 nm; Figure 2C); thus, particles appeared to be physically stable, and no aggregation issues were observed after mal-tNTA conjugation.

#### Attachment of H1 to E2

Our model HA antigen, H1 from A/California/7/2009 (Sino Biological), was His-tagged at the C-terminus, expressed in HEK293T cells, and lacked the transmembrane domain and cytoplasmic tail of native hemagglutinin. The protein also lacks an engineered trimerization domain, and size exclusion chromatography (SEC) comparing the elution profiles of this soluble H1 shows elution profiles consistent with previously reported monomers of H1^72,73^ and other HA subtypes (H3, H5),^72−75^ rather than their trimers (Figure SI-4). Although HA trimers may present quaternary epitopes not found in monomers and thereby elicit antibodies from a broader repertoire of B cell clones,^76,77^ we aim to construct H1–E2 using HA monomers to facilitate synthesis, which is an important consideration for future nanoparticle vaccine scale-up. Moreover, we have also published IgG cross-reactivity profiles against different drift variants for mice that were administered monomeric or trimeric H5; we found that these datasets are highly correlated (*R*^2^ = 0.92), suggesting the immunogenicity of monomeric HA is broadly overlapping with trimeric HA.^78^

To attach the His-tagged H1 to E2, we chelated tNTA-E2 with Ni(II) and incubated it with the His_6_-tagged H1. The H1–E2 product was then separated from unbound H1 by size exclusion chromatography (SEC), resulting in protein elution within two distinct peaks (Figure 2A,B). SDS-PAGE analysis of each of the fractions from the SEC showed that, as expected based on size, the first peak contained E2 nanoparticles with attached H1 and the second peak was free, unbound H1 (Figure SI-5). It should be noted that the tNTA-H1 interaction with the hexahistidine is a coordination, not a covalent, bond and therefore leads to the appearance of separate H1, E2, and tNTA-E2 subunit bands on the SDS-PAGE denaturing gel (Figure 2A, lane 4; Figure SI-5). The H1/E2 binding ratio was calculated from the SEC chromatograms based on the area under the curve as described in the Materials and Methods section. The average ratio of H1 bound per E2 nanoparticle was determined to be 13.1 ± 1.0 and was consistent with estimations based on SDS-PAGE band intensities. This protein assembly yielded an intact H1–E2 particle with an average hydrodynamic diameter of approximately 38.2 ± 1.7 nm (Figure 2C), ca. 9–10 nm larger than the sizes for E2 alone (E279C variant) and tNTA-E2 (Figure 2C). This H1–E2 size is within the range of the ideal size range for lymph node retention times and dendritic cell uptake.^16−20,64^ Dynamic light scattering (DLS) data also did not show evidence of protein aggregation after H1 attachment to the nanoparticle. Transmission electron microscopy of H1–E2 further confirms the intact monodispersed nature of the nanoparticles reported by DLS and the assembly of particles consistent with a hollow dodecahedral cage structure (Figure 2D).

3D protein modeling using ChimeraX shows that on the surface of a 60-mer E2 nanoparticle, the location of the cysteines at position 279 is clustered in a trimeric configuration, with neighboring cysteines estimated to be only ∼3 nm apart (Figure SI-6). Based on modeling, monomeric HA is estimated to have a diameter of ∼3.0 nm. Therefore, it is likely that steric hindrance between HA monomers limits the number of proteins that could associate with the tNTA-functionalized cysteines. Although the resulting average attachment ratio is below the theoretical maximum, this value is close to the observed conjugation of another model protein, green fluorescent protein (GFP), to E2 nanoparticles using the same tNTA/His-tag strategy (Methods in the Supporting Information, Figure SI-7). In examining the attachment of GFP to E2 using this tNTA approach, a molar excess of GFP was reacted with E2 which resulted in ∼9 GFP attached to the surface of E2. We speculate that the theoretical maximum number of 60 H1 on a nanoparticle may not be necessary for a vaccine formulation, since B cell receptor cross-linking has an ideal antigen spacing of 5–10 nm,^8,20,66^ which is above the distance of neighboring cysteines on this platform (Figure SI-6).

### Immunogenicity of H1 Is Enhanced by Conjugation to E2, with or without MPLA

**Figure 3:**
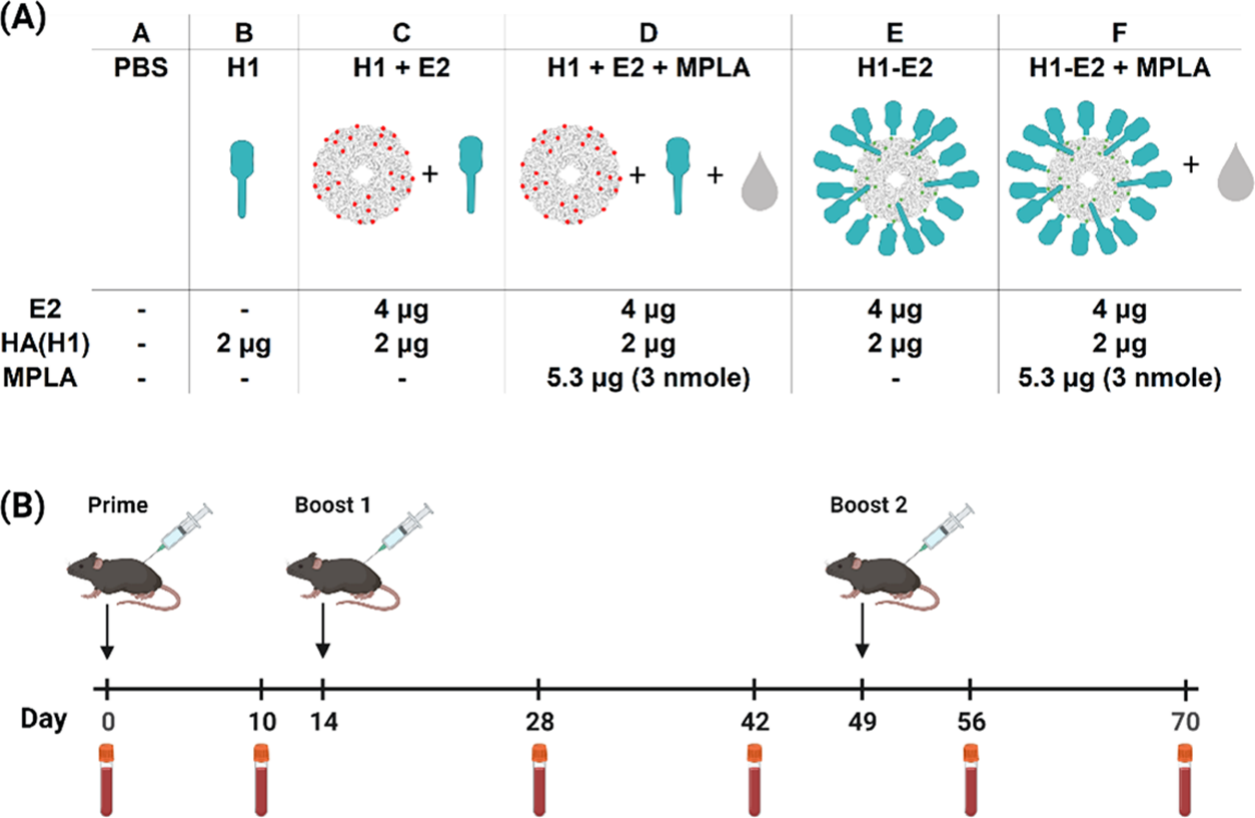
Vaccine groups and immunization schedule. (A) Table summarizing the vaccine groups and dose amounts per injection. Group (A) PBS control; group (B) Hemagglutinin (H1); group (C) E2 nanoparticle and H1 (unconjugated); group (D) E2 nanoparticle and H1 (unconjugated), with MPLA (TLR4 agonist); group (E) H1–E2 (conjugated); group (F) H1–E2 (conjugated) with MPLA. (B). Timeline of immunizations and plasma collection.

**Figure 4:**
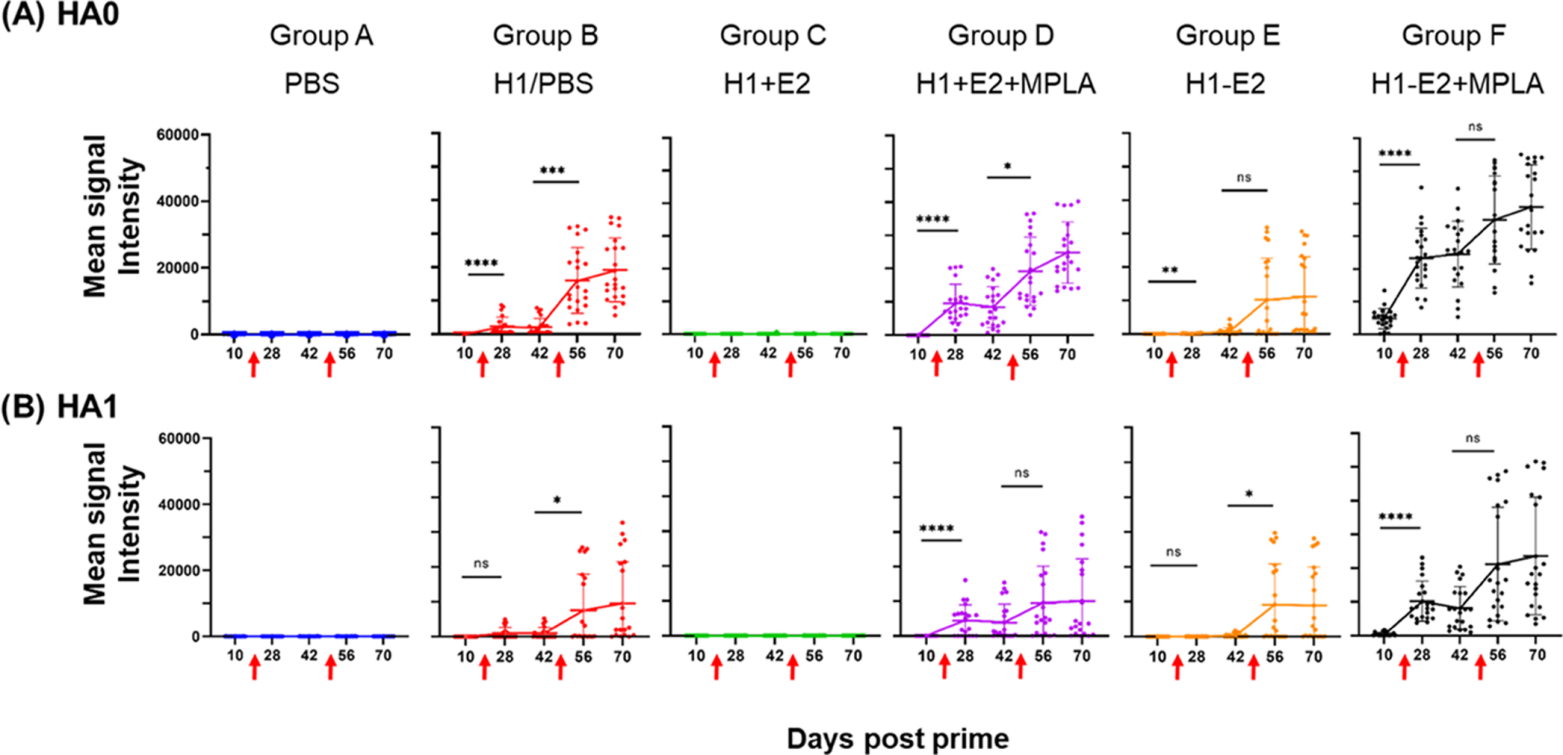
H1-specific IgG profiling by protein microarray. Six groups of five C57BL/6 mice (Groups A–F) were administered different formulations as indicated and boosted on d14 and d49 (red arrows). Array data are shown as dots plots of IgG signal intensities at different time points post-prime against H1 variants displayed on an influenza virus HA protein microarray; each dot represents an individual H1 variant (mean of 5 mice) with lines connecting the means (±SD error bars). (A) HA0, full-length H1 (*N* = 21 variants); (B) HA1 fragment of H1 (*N* = 21 variants). Variants of HA0 and HA1 were utilized and reflect those listed in Supporting Information, Table S1 and Figure SI-10. One-way analysis of variance (ANOVA) (nonparametric) comparisons using a Kruskal–Wallis test were made between both pre- and post-boost time points: ****p < 0.0001, ***p < 0.001, **p < 0.01, *p < 0.05 Abbreviations: PBS, phosphate-buffered saline; H1 + E2, unconjugated H1 and E2 NPs; H1–E2, conjugated H1 and E2 NPs, MPLA, monophosphoryl lipid A.

We then examined the immunogenicity of our H1–E2 nanoparticles in mice *in vivo*, following the vaccine groups and immunization schedule summarized in Figure 3. Plasma samples collected at regular intervals were probed for IgG breadth using HA protein microarrays (Figures 4 and SI-8). IgG reactivity toward full-length (HA0) proteins is shown in Figure 4A. Phosphate-buffered saline (PBS) controls (Group A) failed to produce H1-specific IgG at any time point, as expected. H1 (Group B) was antigenic in the absence of an adjuvant, although required two boosts to generate a broad response across different H1 variants. Conjugation of H1 to E2 NPs (H1–E2, Group E) did not enhance immunogenicity unless also administered with MPLA (Group F). Inclusion of MPLA to the H1–E2 conjugate also dramatically accelerated the response such that IgG was detected on day 10 (after a single dose), with a significant increase in magnitude after the first boost, and a further increase after the second boost. MPLA also enhanced the magnitude of the response to unconjugated H1 (Group D) although signals were lower than when conjugated (Group F). Interestingly, Group C, which received unconjugated H1 and E2 without MPLA, never induced H1-specific antibodies at any point during the study. Since H1 alone was able to induce IgG, there appears to be a suppressive function of E2 when admixed with H1. This suppressive effect does not appear to be unique to H1, as it was also observed when mice were immunized with a different protein antigen (CBU1910 from *Coxiella burnetii*) mixed with E2; however, the suppression that is observed for CBU1910 appears to be at a lower extent (Figure SI-9). The reasons for this are currently unclear but seem to be overcome by the inclusion of MPLA (as can be seen in Group D). At no point in the study did any animals exhibit noticeable adverse reactions, including weight loss to any of the formulations (data not shown).

IgG reactivity toward HA1 fragments is shown in Figure 4B. HA1 contains the variable head domain and part of the conserved stem domain. Thus, cross-reactivity for HA1 is more stringent than for the whole HA0 protein since HA1 contains fewer conserved amino acids found in the stem. Overall, the dynamics of the response are similar to that seen against the full-length HA0, although the magnitude of signals for HA1 fragments is lower, consistent with lower sequence identity between HA1 fragments and the immunizing H1 variant. Moreover, the accelerated (d10) response seen against full-length HA0 in Group F was not seen against the HA1 fragment, suggesting IgG against the stem (not encoded in HA1) arise first.

The early (d10) appearance of H1-specific IgG in Group F suggests conjugation of antigen to the NP enhances class switching of H1-specific B cells. Since neither unconjugated H1 with MPLA (Group D) nor conjugated H1–E2 without MPLA (Groups D and E, respectively) shows IgG at d10, the data from Group F indicates physical linkage of H1 to E2 and MPLA are synergistic in the acceleration of the response. This may be owing to the increased size of the H1–E2 complex compared to unconjugated H1, which may lead to improved lymph node retention required for class switching and affinity maturation.^64,79,80^ In addition, the display of H1 on the E2 NP in a repeating manner is conducive for B cell receptor (BCR) cross-linking, which is a requirement for B cell activation.^81^ BCR cross-linking has also recently been shown to play a role in germinal center induction within lymph nodes, a necessary step for achieving affinity maturation and class switching of B cells.^82^ Finally, because the particulate nature of the H1–E2 nanoparticle makes it more likely to be taken up by APCs, we speculate that these cells may have higher levels of H1-derived peptide/MHC complexes on their surface leading to enhanced T cell activation.^83,84^ Of note, activated CD4 “helper” T cells can then contribute to T cell-dependent maturation of B cells to elicit higher IgG titers. It is suspected that one or a combination of the above mechanisms are contributing to the accelerated and increased magnitude of signals observed in Group F.

### H1–E2 Nanoparticle Immunizations Elicited an IgG1/IgG2c Balanced Antibody Response

**Figure 5:**
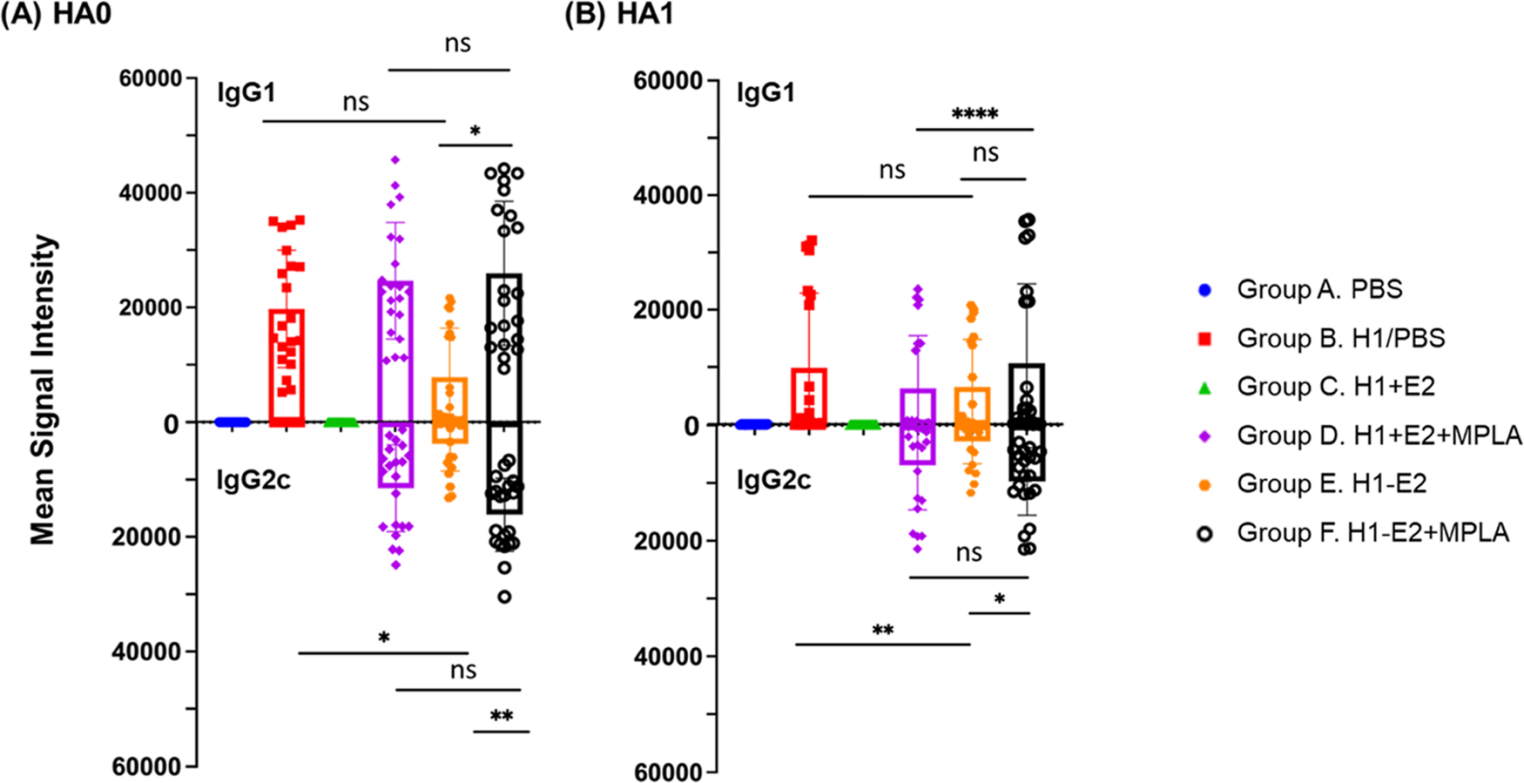
Attachment of H1 to E2 (H1–E2) modulates the immune response to H1 toward a more balanced IgG1/IgG2c antibody response. Box and whisker plots with each dot representing an individual H1 variant (means ± SD of *n* = 5 mice) after probing d70 samples on HA protein microarrays followed by IgG1 and IgG2c-specific secondary antibodies. (A) HA0, full-length H1, (B) HA1 fragment of H1. 21 variants of HA0 and 21 variants of HA1 were utilized and reflect the H1 variants listed in Supporting Information, Table S1 and Figure SI-10. One-way ANOVA (nonparametric) comparisons using a Kruskal–Wallis test for means of the data were performed between groups as shown, ****p < 0.0001, ***p < 0.001, **p < 0.01, *p < 0.05. Abbreviations: PBS, phosphate-buffered saline; H1 + E2, unconjugated H1 and E2 NPs; H1–E2, conjugated H1 and E2 NPs, MPLA, monophosphoryl lipid A.

Endpoint plasma (d70) was also probed for H1-specific IgG1 and IgG2c using isotype-specific secondary antibodies (Figure 5). Group B (unadjuvanted H1) defaults to a strongly IgG1-polarized response. In contrast, Group D (unconjugated E2 + H1 with MPLA) and Group F (conjugated H1–E2 with MPLA) elicited a balanced IgG1 and IgG2c response, with Group F eliciting marginally higher IgG2c signals than group D. Group E (H1–E2 without MPLA) also induced a balanced response, although the magnitude of the signals was significantly lower in the absence of MPLA.

IgG1/IgG2 subtyping is frequently used as a surrogate marker for Th2 and Th1 functionality, respectively.^85−87^ In previous studies we showed the capacity of E2 NP to elicit antitumor immunity when conjugated with tumor peptide antigens and administered with CpG (a TLR9 agonist) as an adjuvant.^28,62,63^ The Th1-skewing property of CpG is well known.^88^ In the present study, the H1–E2 conjugate in the absence of MPLA (Group E) was able to elicit modest IgG2c, while H1 alone could not, which suggests that the E2 NP itself may have some inherent Th1-biasing properties. This is a novel finding and is significant as many FDA-approved human vaccines adjuvanted with alum (aluminum hydroxide salts) are biased toward stimulating Th2 immunity. Although neutralizing antibody responses have conventionally been the focus of evaluating influenza vaccine efficacy, it is now clear that Th1 and cell-mediated responses are also important for protection against influenza.^89,90^

Unlike B cells, which are confined to recognizing structural, often highly variable, antigens on the surface of viruses, T cells can recognize epitopes found within nonstructural antigens expressed in infected cells, and which in many cases are highly conserved between variants. This potentially high sequence conservation makes T cell epitopes promising vaccine antigens, particularly for pathogens such as influenza which has the capacity to undergo antigenic drift. CD8 T cells, which act by killing infected cells that present pathogen-associated peptide epitopes on MHCI, have long been demonstrated to have the capacity to react against heterosubtypic influenza strains^91,92^ and their role in controlling symptomatic infection is well documented.^93,94^ Although the design of E2 nanoparticle-based cancer vaccines with tumor-associated antigens has been demonstrated to elicit a CD8 cytotoxic response,^62,63^ the utility of E2 for inducing CD8 to whole protein antigen is still under investigation. Our data does show, however, the ability of our E2 NP to skew IgG responses toward IgG2c, suggesting a stronger Th1 CD4 cell-mediated response, which may have benefits in the context of influenza vaccine design.^95,96^

### H1–E2 Conjugated NPs Elicited Broader Homosubtypic and Heterosubtypic Cross-Reactivity than Unconjugated H1

**Figure 6:**
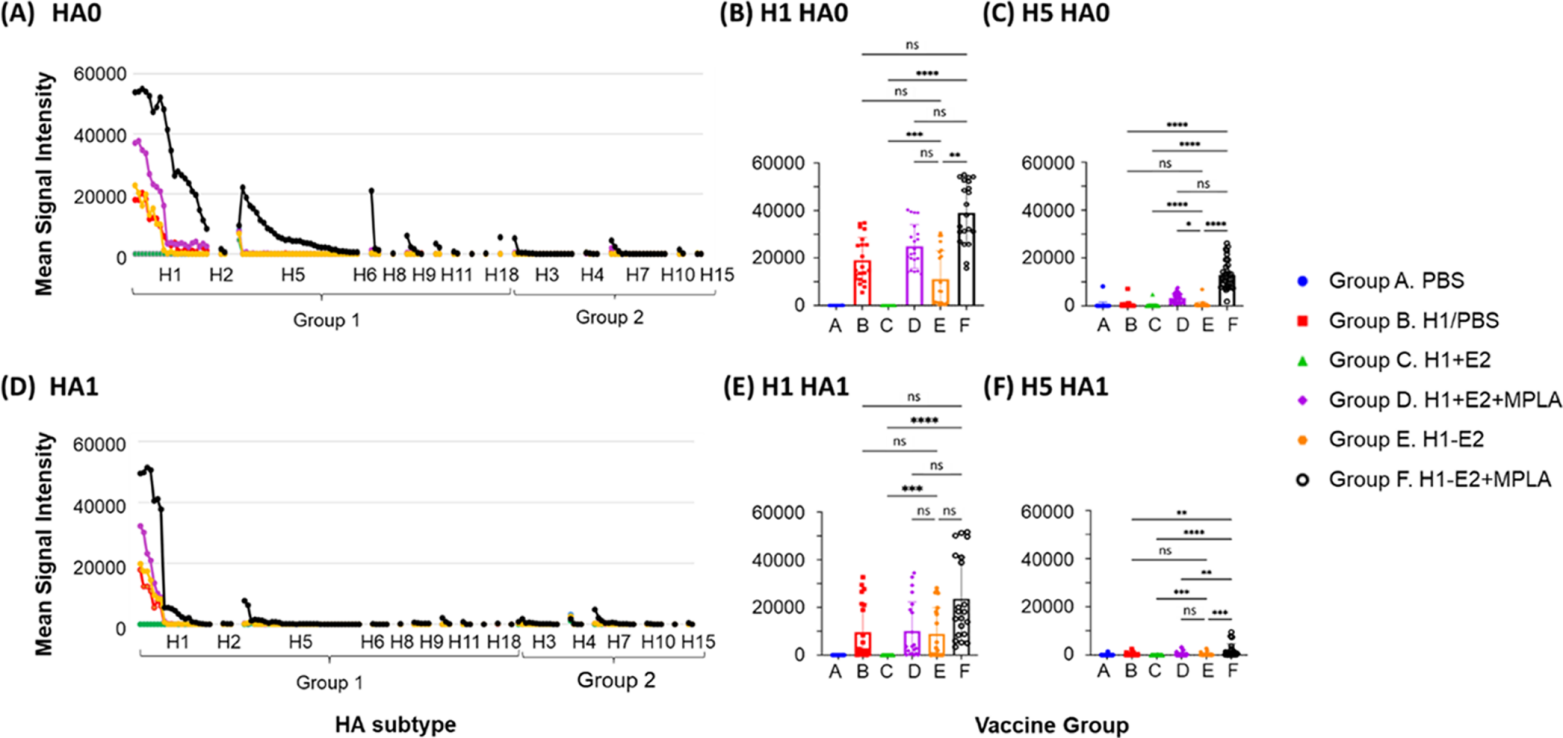
Attachment of H1 to E2 nanoparticle (H1–E2) engenders homosubtypic and heterosubtypic cross-reactivity that is enhanced by MPLA. (A, D) Day 70 plasma IgG profiles against full-length (HA0) and HA1 fragments, respectively, as measured by protein microarray. Each spot corresponds to an individual arrayed protein (mean of 5 mice). The arrayed proteins (horizontal axis) are arranged by phylogenetic group and ranked by descending signal intensity. (B, C) Box plots of H1 and H5 full-length (HA0) array data. (E, F) Box plots of H1 and H5 HA1 fragment array data, each showing one-way ANOVA (nonparametric) comparisons using a Kruskal–Wallis test for means as indicated; ****p < 0.0001, ***p < 0.001, **p < 0.01, *p < 0.05.

Subtype cross-reactivity on the array is a potential correlate of the breadth of the response induced by a vaccine. The head region of HA, encoded in HA1, is the most variable region found between influenza variants and contains epitopes recognized by neutralizing antibodies. Mutations within neutralizing antibody epitopes (located predominantly in the immunodominant head of HA) lead to immune selection of variants able to escape antibody neutralization and the emergence of novel variants.^97^ More conserved regions, located in both the HA1 and HA2 domains, that play functional roles in receptor binding and membrane fusion, respectively, are widely considered as prime targets for eliciting broad or cross-protective immunity. However, such vulnerabilities are often masked by glycans^98−101^ and may be inaccessible to antibodies or subdominant in the response, making them unsatisfactory targets for vaccination. Here we demonstrate that loading H1 on E2 NPs enhances antibody responses toward both of head and stem regions of different influenza subtypes. Shown in Figure 6 are IgG profiles for day 70 plasma. (A list of the HA proteins printed on the microarray and resulting data are given in Supporting Information Table S1.) The plots in Figure 6A show the signals for each vaccine group (mean of 5 mice) against all HA0 (full-length) HAs printed on the array, spanning HA subtypes 1 through 18 (horizontal axis), organized by phylogenetic group. The data for individual full-length (HA0) H1 and H5 variants are also shown in the box plots in Figure 6B,C, respectively. H1 and H5 both belong to phylogenetic group 1 and have ∼63% amino acid sequence identity. While vaccine Groups B, D, E, and F were all able to elicit homosubtypic cross-reactivity (i.e., to the H1 variants) only Group F (H1–E2 conjugate with MPLA) elicited detectable heterosubtypic responses for H5 and other Group 1 subtypes. Overall, Group F elicited both the highest homo- and heterosubtypic antibody signals.

The plots in Figure 6D show the mean signals for each vaccine group for all HA1 fragments on the array. The HA1 fragment contains the variable head domain of HA and part of the stem. Overall, the same vaccine formulations that induced a homosubtypic cross-reactive response to the full-length (HA0) H1 molecule, also induced a homosubtypic cross-reactive response to the H1 HA1 fragment, although the breadth was narrower. Similarly, Group F, which was the only group able to induce significant heterosubtypic cross-reactivity for full-length H5, also induced a modest response to H5 HA1 fragments, although again the breadth was reduced. The data for individual HA1 fragments of H1 (homosubtypic cross-reactivity) and H5 (heterosubtypic cross-reactivity) are shown in the box plots in Figure 6E,F, respectively. These representations of the data emphasize that the breadth of both homo- and heterosubtypic responses are broader for the full-length HA0 compared to the HA1 fragment.

This relatively broader response to full length (HA0) compared to HA1 fragment by protein microarray (Figure 6) has been seen in other studies where the breadth of the response induced by adjuvanted HA was examined using the microarray platform.^78,102^ One possibility is the stem is immunodominant over the head, at least when recombinant HA protein is delivered in adjuvant. This contrasts with the response to natural infection where the head is usually immunodominant.^103^ We speculate the membrane distal head is readily accessible to antibody on the virion surface, whereas the stem might be relatively less accessible to antibody, accounting for the immunodominance of the head during natural infection. In contrast, administration of recombinant HA protein in adjuvant may allow the stem to be more available for antibody recognition. Regardless of the precise mechanism, this is significant because, in contrast to the variable head domain, the stem is relatively well conserved and a vaccine strategy able to drive the response toward the conserved stem may offer a path to a more broadly protective vaccine compared to current vaccine approaches.^104^ We are also aware it is possible the immunodominance of the stem in this study may be because HA1 adopts a more authentic conformation in the array platform when assembled in the full-length (HA0) molecule rather than as an HA1 fragment. Monoclonal antibodies (mAbs) that are usually used to probe for correct conformation of HA typically recognize the stem rather than the head; conversely, HA1-binding mAbs (e.g., those that inhibit virus-mediated hemagglutination) generally bind to linear epitopes and are not conformation-dependent. Nevertheless, we did identify two conformation-sensitive mAbs that bind H1 HA1 on the array that lost reactivity against denatured antigen,^102^ supporting the notion that HA1 is correctly folded on the array. However, further studies to establish whether the administration of recombinant HA in adjuvant helps overcome immunodominance of the head are warranted.

Homosubtypic cross-reactivity of antibodies generated by the H1 (A/California/7/2009) vaccine and other H1 drift variants is mediated by B cell clones that recognize antigenically similar epitopes. Although not defined here, these epitopes will map to conserved amino acids shared by multiple variants and are typically regions of the HA protein required for structural stability, receptor-mediated attachment, or membrane fusion.^105^ Homosubtypic cross-reactivity elected by the H1–E2 vaccine reported here is significant, as it may offer a path to providing protection against drift variants. Current seasonal influenza vaccines, manufactured predominantly from inactivated influenza virus and detergent extracted to enrich for the membrane HA and NA molecules, elicit antibodies that are highly specific to the immunizing variant. Consequently, seasonal vaccines need to be revised each year in response to antigenic drift, i.e., the process by which the circulating viruses (currently H1N1 and H3N2) accumulate mutations within the neutralizing epitopes under selective pressure from antibodies. Although the breadth of the response can be broadened using adjuvants^102^ these are not routinely used for seasonal influenza.

Seasonal influenza vaccines provide negligible heterosubtypic cross-reactivity. Here we show that among the formulations tested, the conjugated H1–E2 with MPLA induced cross-reactivity for H5. Avian influenza H5N1 is endemic in wild birds and frequently causes outbreaks in domestic poultry; it is also well known for causing zoonotic infections in humans.^106^ Indeed, the risk of pandemics caused by H5N1 has prompted the stockpiling of H5N1-based vaccines as part of the US Government’s Pandemic Influenza Vaccine Stockpiling Program. A vaccine able to provide broader protection, achieved through the use of improved delivery systems such as adjuvants and/or nanoparticles, would reduce the need for annual reformulations and may lead to vaccines against both seasonal and pandemic influenzas, or ideally across all subtypes (so-called “universal” influenza vaccines).

## Conclusions

A model antigen (H1 hemagglutinin from influenza) has been successfully conjugated to the E2 NP using a new tris-NTA chemical linker that utilizes polyhistidine tags engineered into recombinant proteins. Unlike many conventional protein–protein linkers, this strategy enables the attachment of protein antigens onto the surface of a vaccine nanoparticle platform in a defined orientation, which can potentially increase B-cell activation. We tested the antibody responses after administration with vaccine nanoparticles that were fabricated using this approach. In immunogenicity studies in mice where H1–E2 was compared to unconjugated H1, the conjugate elicited a more balanced IgG1/IgG2c response to H1, compared to the strongly polarized IgG1 response seen against H1 alone, showing the E2 particle may have an inherent Th1-biasing property. Administration of the H1–E2 conjugate with MPLA significantly accelerated the response (with IgG appearing on d10) but not when administered with unconjugated H1, nor when H1–E2 conjugate was administered in the absence of MPLA, suggesting NP-mediated delivery of antigen and MPLA signaling synergize to accelerate the response. While several formulations tested engendered homosubtypic cross-reactivity, only the conjugated H1–E2 NP with MPLA induced significant heterosubtypic cross-reactivity, a favorable characteristic for vaccine designs that may protect against drift variants. Our tris-NTA/His-tag conjugation strategy is applicable to other protein antigens and should broaden the utility of the E2 NP as a delivery vehicle for other human pathogens.

## Materials and Methods

### Materials

Chemical reagents were purchased from Fisher Scientific, Sigma-Aldrich, Acros Organics, Iris Biotech, or TCI Pharmaceuticals unless otherwise noted. Phosphate buffer used for reactions in this study comprised 50 mM KH_2_PO4 and 100 mM NaCl at a pH 7.4. Phosphate-buffered saline (PBS) used for *in vivo* studies was purchased from Gibco. 4-(2-Hydroxyethyl)-1-piperazineethanesulfonic acid (HEPES) buffers used in this study included 20 mM HEPES with 100 mM NaCl or 360 mM NaCl at a pH 7.3. Aqueous stock solutions of NiCl_2_ were made at 500 mM. Stock solutions of mal-tNTA were made at 4 mg/mL in dimethylformamide (DMF). All HA proteins used in this investigation, including H1 for conjugating to E2 (variant A/California/07/2009, H1N1) and HA variants used in the protein arrays, were purchased from Sino Biological, and catalog numbers are listed in Supporting Information, Table S1.

### Synthesis of Maleimido Cyclic tris-NTA (mal-tNTA)

To generate mal-tNTA (5), the synthesis was performed following the route described in Scheme 1. The synthesis of *t-*butyl-protected tris-NTA-NH (4) was first performed as previously described^53^ and these steps, with minor modifications, are detailed in Supporting Information. To produce *t-*butyl-protected tris-NTA from (4), maleimido-propionic acid (27 mg, 0.16 mmol) and *N*,*N*,*N*′,*N*′-tetramethyl-*O*-(1*H*-benzotriazol-1-yl)uronium hexafluorophosphate (HBTU, 61 mg, 0.16 mmol) were dissolved in DMF (9.5 mL) and *N*,*N*-diisopropylethylamine (DIPEA, 0.5 mL). After 5 min, *t*-butyl-protected tris-NTA-NH (151 mg, 0.11 mmol) was added and the reaction was stirred overnight. The solvent was evaporated, and the product was purified by flash chromatography. Column conditions were: 40 g silica gel column; mobile phase A: hexanes; mobile phase B: ethyl acetate. This was run at a gradient condition of mobile phase B: 0–4 min 0% B, 4–12 min 100% B ramp, 12–20 min 100% B. The product eluted at 13 min. The fractions containing product were collected, and the solvent was removed by rotary evaporation and dried over high vacuum. The product was recovered and analyzed by electrospray ionization mass spectrometry (ESI-MS) (110 mg, 66% yield). ESI was performed on a Waters LCT ESI MS with flow injection at 0.1 mL/min in 100% MeOH. Predicted [M + Na]^+^: 1613.9 *m*/*z* Observed [M + Na]^+^: 1612.9 *m*/*z*.

*t*-Butyl-protected tris-NTA-mal (109 mg, 0.069 mmol) was then dissolved in 95% trifluoroacetic acid (TFA) in water (5 mL) and stirred for 2 h. The TFA was removed by rotary evaporation, and the residue was added to 40 mL of cold diethyl ether to precipitate the product. The mixture was centrifuged to recover the product pellet. The pellet was dissolved in 50% water/ACN, sterile filtered through a nylon 0.22 μm filter, and lyophilized. The product (5) was weighed and analyzed by LC–MS in water/acetonitrile with 0.1% formic acid (53 mg, 71% yield). LC–MS was performed on a Waters LC–MS with QDA detector with Hclass UPLC with a water/acetonitrile 0.1% formic acid solvent system. Predicted [M + H]^+^: 1087.4 *m*/*z* Observed [M + H]^+^: 1087.8 *m*/*z*.

### E2 Nanoparticle Expression and Purification

Expression and purification of the E2 protein nanoparticle were performed as previously described.^57,107^ In this study, the E2 mutant E279C was used. This mutant has the native glutamic acid at position 279, which resides on the exterior surface of the nanoparticle, replaced with a cysteine residue enabling the conjugation of the mal-tNTA (and subsequently, HA antigen) on the thiol.^107^ Briefly, BL21(DE3) *Escherichia coli* cells containing the plasmid with the E279C gene were grown in LB media with ampicillin, and protein expression was induced with IPTG. Soluble cell lysates were applied to a HiPrep Q Sepharose anion exchange column (GE Healthcare) followed by a Superose 6 size exclusion column (SEC, GE Healthcare) for purification, with 1 mM dithiothreitol (DTT) added to all lysis, purification, and storage buffers to reduce disulfide bonds and prevent protein cross-linking of the cysteines. The hydrodynamic diameter of the purified protein nanoparticles was analyzed by dynamic light scattering (DLS; Zetasizer Nano-ZS ZEN3600, Malvern). Electrospray ionization mass spectrometry (Xevo G2-XS Qtof) and SDS-PAGE confirmed molecular weight and purity. Final protein preparations were stored in 50 mM potassium phosphate at pH 7.4 with 100 mM NaCl and 1 mM DTT at 4 °C for short-term and −80 °C for long-term storage. Lipopolysaccharide was removed using Triton X-114 (Sigma), residual surfactant was removed with detergent removal spin columns (Pierce), and low endotoxin levels were confirmed with a LAL ToxinSensor kit (Genscript).^61^ Protein concentrations were quantified by bicinchoninic acid (BCA) (Pierce, Thermo Fisher Scientific).

### Conjugation of Protein Antigen to Protein Nanoparticle

The overall strategy for conjugating the hemagglutinin (HA) antigen to E2 is shown in Figure 1. The attachment is mediated by a hexahistidine/Ni-NTA interaction, with the histidine tag on HA and tNTA on E2.

#### Conjugation of mal-tNTA to E2

To remove DTT from purified E2, the E2 nanoparticles were passed through a 0.5 mL 40 kDa molecular weight cutoff Zeba spin desalting column (Thermo Fisher Scientific) to exchange with HEPES buffer (20 mM HEPES, 100 mM NaCl, pH 7.3) according to manufacturer instructions. An 8.5× molar excess of tris(2-carboxyethyl)phosphine (TCEP) (Thermo Fisher Scientific) was added and incubated with the E2 (30–45 min at room temperature), followed by addition and incubation with a 10× molar excess of mal-tNTA (1–2 h at room temperature, then at 4 °C overnight) (final DMF concentration did not exceed 10% (v/v)). A buffer exchange was performed with a desalting column using 20 mM HEPES, 100 mM NaCl to remove unreacted mal-tNTA, DMF, and TCEP. Conjugation efficiency of the mal-tNTA to E2 and characterization were performed via SDS-PAGE and ESI-MS (Xevo G2-XS QTof). Protein concentration and hydrodynamic diameters and protein concentrations were measured via BCA and DLS, respectively.

#### Attachment of His_6_-Tagged Hemagglutinin (HA) to E2 Nanoparticle

A 10× molar excess of NiCl_2_ in aqueous solution was added to the tNTA-E2 and incubated for 2 h at room temperature with gentle shaking. Unchelated NiCl_2_ was removed and buffer exchange (20 mM HEPES, 360 mM NaCl, pH 7.3) was performed by a spin desalting column. A C-terminally His_6_-tagged HA monomer lacking its native transmembrane domain and cytoplasmic tail from A/California/07/2009 (H1N1) (Sino Biological, Inc.; reconstituted at 1 mg/mL in water; GenBank protein accession #ACP41105.1) was added to Ni-tNTA-E2 at a 0.3:1 ratio of H1/E2 monomer and incubated at room temperature with gentle shaking for 2 h. The mixture was filtered with a 0.22 μm poly(vinylidene difluoride) (PVDF) membrane and separated with a Superose 6 analytical size exclusion column (SEC) column on ÄKTA FPLC (Cytiva/GE Healthcare) to remove unbound H1. Fractions were evaluated with an SDS-PAGE gel and stained with a Pierce Silver Stain Kit (Thermo Fisher Scientific) to identify the fractions containing H1 attached to E2 nanoparticles (H1–E2) vs unbound H1. Conjugation efficiency of H1 to E2 was estimated using the SEC chromatographs that showed unbound H1 (peak 2) and conjugated H1–E2 (peak 1) (Figure 2B). In brief, we calculated the concentration of unreacted H1 from the area under the curve (AUC) using the volume and *A* = ϵ*bC* (where *A* = absorbance, ϵ = molar extinction coefficient of H1, *b* = path length, and *C* = molar concentration of unreacted H1). The extinction coefficient of H1 was estimated using Expasy ProtParam (88,240 M^–1^ cm^–1^).^108^ Mole balances of H1 and E2 related the total amount of H1 and E2 input into the conjugation reaction (respectively) with the amounts after reaction, which enabled the determination of ratio of H1 bound per E2 nanoparticle (*n* = 9 independent conjugation batches).

### Transmission Electron Microscopy

Five microliters of the nanoparticles at approximately 0.015 mg/mL were applied to glow-discharged carbon-coated grids and negatively stained with a saturated ammonium molybdate solution. The sample grids were examined with a JEM-2100F transmission electron microscope (JEOL) equipped with a OneView CCD (Gatan).

### Protein Modeling

ChimeraX was used to model E2 nanoparticle (PDB code: 1b5s) and H1 monomer (PDB code: 3ztn), estimate protein dimensions, and generate protein graphics.^109,110^ Protein dimensions were measured using ChimeraX’s “distance” command and RCSB Protein Data Bank’s (PDB’s) distance tool. Two amino acids on opposite sides of the H1 stem region were selected, and their distance was recorded. Multiple pairs of amino acids were analyzed and an average distance for the width of H1 was calculated. A similar procedure was done to determine the distance between cysteines on the E2 NP.

### Immunizations with H1–E2 Nanoparticles

All animal studies were carried out in accordance with protocols approved by the Institute for Animal Care and Use Committee (IACUC) at the University of California, Irvine, and by the Animal Care and Use Review Office (ACURO) of the U.S. Army Medical Research and Materiel Command (USAMRMC). Six groups (*N* = 5 per group) of 6- to 8-week-old C57BL/6 female mice (Charles River) were administered 100 μl vaccine formulations in phosphate-buffered saline (PBS, Gibco) (Figure 3A) via the subcutaneous route (base of the tail) according to the schedule shown in Figure 3B. We examined the effects of immunizing with H1 bound and unbound to the E2 nanoparticle, with and without the TLR4 agonist, monophosphoryl lipid A (MPLA) (Avanti Polar Lipids, Inc.). PBS and H1 in PBS served as control groups. H1 and E2 nanoparticles were administered at 2 and 4 μg/dose, respectively. Since MPLA has limited solubility in aqueous solution, MPLA was integrated into 1,2-dioleoyl-*sn*-glycero-3-phospho-(1'-*rac*-glycerol) (DOPG) liposomes (an inert co-lipid) at a 1:5 molar ratio. The mice were primed via the s.c. route (base of tail) and boosted with identical formulations via the same route on days 14 and 49. The mice were weighed daily for approximately 2 weeks after each injection and monitored for any changes in behavior or appearance. On days 0, 10, 28, 42, and 56, blood was collected via cheek vein bleed. Blood was collected via cardiac puncture on day 70, the experimental end point.

### Antibody Profiling by Influenza Protein Microarray

The construction and probing methology of the influenza protein microarray used for the study has been reported previously.^111^ Briefly, over 200 recombinant influenza HAs spanning 18 subtypes, expressed in human or insect cells as either HA0 or HA1 molecules with a C-terminal His-tag, were purchased from Sino Biological Inc. and printed as described.^111^ The array content and raw data are shown in Supporting Information, Table S1. Plasma samples were incubated with rehydrated arrays at 4°C overnight and washed in tris-buffered saline (TBS) containing 0.05% Tween 20 (T-TBS). Bound IgG was detected using biotinylated anti-mouse IgG (Jackson ImmunoResearch; Cat No. 115-068-071) and visualized after washing using streptavidin-conjugated Qdot-800 (Life Technologies; Cat. No. Q10173MP). For IgG subtyping, anti-mouse IgG1-Alexa Fluor 647 or IgG2c-Alexa Fluor 555 (Southern Biotech; Cat. Nos. 1073-31 and 1077-32) were used. After washing and drying, images were acquired using the ArrayCAM imaging system (Grace Bio-Labs Inc., Bend, OR).

### Statistical Analyses

Data describing nanoparticle characterization (e.g., hydrodynamic diameter, antigen/nanoparticle ratios, mass spectrometry molecular weights) are presented as mean ± standard deviation (SD) of at least three independent experiments (*n* ≥ 3), unless otherwise noted. Protein microarray data from immunized mice sera was compiled in dot plots of signal intensities for each antigen (mean ± SD for each vaccine group) generated in Prism version 9.3.1 (GraphPad, LA Jolla, CA). One-way ANOVAs were performed using a Kruskal–Wallis multiple comparison test (Dunn’s multiple comparison) in Prism; a *P* value of <0.05 was considered statistically significant.

## Supporting Information Available

Additional experimental materials and methods including mal-tNTA synthesis; structure of HA (Figure SI-1); LC–MS of mal-tNTA (Figure SI-2); mass spectrometry of tNTA-E2 (Figure SI-3); size exclusion chromatography (SEC) of H1 (Figure SI-4); SDS-PAGE of H1–E2 SEC (Figure SI-5); E2 (E279C) ChimeraX-generated image with measurements (Figure SI-6); GFP attachment to E2 (Figure SI-7); protein microarrays of immunized mice sera (Figure SI-8); immunization study of E2 and protein antigen, CBU1910, admixed (Figure SI-9); and list of H1 HA0 and H1 HA1 variants on the protein microarrays for antibody profiling (Figure SI-10) (PDF)
Influenza protein microarray contents and raw data values from IgG, IgG1, and IgG2 probing (Table SI-1) (XLSX)

## References

[ref1] Park Y. W.; Kim Y. H.; Jung H. U.; Jeongl O. S.; Hong E. J.; Kim H.; Lee J. I. Comparison of antigenic mutation during egg and cell passage cultivation of H3N2 influenza virus. Clin. Exp. Vaccine Res. 2020, 9, 56–63. 10.7774/cevr.2020.9.1.56. 32095441 PMC7024727

[ref2] Wu N. C.; Zost S. J.; Thompson A. J.; Oyen D.; Nycholat C. M.; McBride R.; Paulson J. C.; Hensley S. E.; Wilson I. A. A structural explanation for the low effectiveness of the seasonal influenza H3N2 vaccine. PLoS Pathog. 2017, 13, e1006682 10.1371/journal.ppat.1006682. 29059230 PMC5667890

[ref3] Wang W.; Alvarado-Facundo E.; Vassell R.; Collins L.; Colombo R. E.; Ganesan A.; Geaney C.; Hrncir D.; Lalani T.; Markelz A. E.; Maves R. C.; McClenathan B.; Mende K.; Richard S. A.; Schofield C.; Seshadri S.; Spooner C.; Utz G. C.; Warkentien T. E.; Levine M.; Coles C. L.; Burgess T. H.; Eichelberger M.; Weiss C. D. Comparison of A(H3N2) Neutralizing Antibody Responses Elicited by 2018-2019 Season Quadrivalent Influenza Vaccines Derived from Eggs, Cells, and Recombinant Hemagglutinin. Clin. Infect. Dis. 2021, 73, E4312–E4320. 10.1093/cid/ciaa1352. 32898271

[ref4] Garretson T. A.; Petrie J. G.; Martin E. T.; Monto A. S.; Hensley S. E. Identification of human vaccinees that possess antibodies targeting the egg-adapted hemagglutinin receptor binding site of an H1N1 influenza vaccine strain. Vaccine 2018, 36, 4095–4101. 10.1016/j.vaccine.2018.05.086. 29861178 PMC5995672

[ref5] Zost S. J.; Parkhouse K.; Gumina M. E.; Kim K.; Perez S. D.; Wilson P. C.; Treanor J. J.; Sant A. J.; Cobey S.; Hensley S. E. Contemporary H3N2 influenza viruses have a glycosylation site that alters binding of antibodies elicited by egg-adapted vaccine strains. Proc. Natl. Acad. Sci. U.S.A. 2017, 114, 12578–12583. 10.1073/pnas.1712377114. 29109276 PMC5703309

[ref6] Sanders B.; Koldijk M.; Schuitemaker H. Inactivated Viral Vaccines. In Vaccine Analysis: Strategies, Principles, and Control; Springer: Berlin, Heidelberg, 2014; pp 45–80.

[ref7] Minor P. D. Live attenuated vaccines: Historical successes and current challenges. Virology 2015, 479–480, 379–392. 10.1016/j.virol.2015.03.032. 25864107

[ref8] Moyle P. M.; Toth I. Modern Subunit Vaccines: Development, Components, and Research Opportunities. ChemMedChem 2013, 8, 360–376. 10.1002/cmdc.201200487. 23316023

[ref9] Tam H. H.; Melo M. B.; Kang M.; Pelet J. M.; Ruda V. M.; Foley M. H.; Hu J. K.; Kumari S.; Crampton J.; Baldeon A. D.; Sanders R. W.; Moore J. P.; Crotty S.; Langer R.; Anderson D. G.; Chakraborty A. K.; Irvine D. J. Sustained antigen availability during germinal center initiation enhances antibody responses to vaccination. Proc. Natl. Acad. Sci. U.S.A. 2016, 113, E6639–E6648. 10.1073/pnas.1606050113. 27702895 PMC5086995

[ref10] Nguyen B.; Tolia N. H. Protein-based antigen presentation platforms for nanoparticle vaccines. npj Vaccines 2021, 6, 11 10.1038/s41541-021-00330-7. 33986287 PMC8119681

[ref11] Nanishi E.; Dowling D. J.; Levy O. Toward precision adjuvants: optimizing science and safety. Curr. Opin. Pediatrics 2020, 32, 125–138. 10.1097/MOP.0000000000000868. PMC6970548 31904601

[ref12] Liu Y. V.; Massare M. J.; Barnard D. L.; Kort T.; Nathan M.; Wang L.; Smith G. Chimeric severe acute respiratory syndrome coronavirus (SARS-CoV) S glycoprotein and influenza matrix 1 efficiently form virus-like particles (VLPs) that protect mice against challenge with SARS-CoV. Vaccine 2011, 29, 6606–6613. 10.1016/j.vaccine.2011.06.111. 21762752 PMC3165014

[ref13] Jardine J.; Julien J. P.; Menis S.; Ota T.; Kalyuzhniy O.; McGuire A.; Sok D.; Huang P. S.; MacPherson S.; Jones M.; Nieusma T.; Mathison J.; Baker D.; Ward A. B.; Burton D. R.; Stamatatos L.; Nemazee D.; Wilson I. A.; Schief W. R. Rational HIV Immunogen Design to Target Specific Germline B Cell Receptors. Science 2013, 340, 711–716. 10.1126/science.1234150. 23539181 PMC3689846

[ref14] Brune K. D.; Leneghan D. B.; Brian I. J.; Ishizuka A. S.; Bachmann M. F.; Draper S. J.; Biswas S.; Howarth M. Plug-and-Display: decoration of Virus-Like Particles via isopeptide bonds for modular immunization. Sci. Rep. 2016, 6, 19234 10.1038/srep19234. 26781591 PMC4725971

[ref15] Lee K. L.; Twyman R. M.; Fiering S.; Steinmetz N. F. Virus-based nanoparticles as platform technologies for modern vaccines. Wiley Interdiscip. Rev.: Nanomed. Nanobiotechnol. 2016, 8, 554–578. 10.1002/wnan.1383. 26782096 PMC5638654

[ref16] Fifis T.; Gamvrellis A.; Crimeen-Irwin B.; Pietersz G. A.; Li J.; Mottram P. L.; McKenzie I. F. C.; Plebanski M. Size-dependent immunogenicity: Therapeutic and protective properties of nano-vaccines against tumors. J. Immunol. 2004, 173, 3148–3154. 10.4049/jimmunol.173.5.3148. 15322175

[ref17] Foged C.; Brodin B.; Frokjaer S.; Sundblad A. Particle size and surface charge affect particle uptake by human dendritic cells in an in vitro model. Int. J. Pharm. 2005, 298, 315–322. 10.1016/j.ijpharm.2005.03.035. 15961266

[ref18] Chaudhuri A.; Battaglia G.; Golestanian R. The effect of interactions on the cellular uptake of nanoparticles. Phys. Biol. 2011, 8, 046002 10.1088/1478-3975/8/4/046002. 21508440

[ref19] Reddy S. T.; Rehor A.; Schmoekel H. G.; Hubbell J. A.; Swartz M. A. In vivo targeting of dendritic cells in lymph nodes with poly(propylene sulfide) nanoparticles. J. Controlled Release 2006, 112, 26–34. 10.1016/j.jconrel.2006.01.006. 16529839

[ref20] Bachmann M. F.; Jennings G. T. Vaccine delivery: a matter of size, geometry, kinetics and molecular patterns. Nat. Rev. Immunol. 2010, 10, 787–796. 10.1038/nri2868. 20948547

[ref21] Jegerlehner A.; Storni T.; Lipowsky G.; Schmid M.; Pumpens P.; Bachmann M. F. Regulation of IgG antibody responses by epitope density and CD21-mediated costimulation. Eur. J. Immunol. 2002, 32, 3305–3314. 10.1002/1521-4141(200211)32:11<3305::AID-IMMU3305>3.0.CO;2-J. 12555676

[ref22] Kato Y.; Abbott R. K.; Freeman B. L.; Haupt S.; Groschel B.; Silva M.; Menis S.; Irvine D. J.; Schief W. R.; Crotty S. Multifaceted Effects of Antigen Valency on B Cell Response Composition and Differentiation In Vivo. Immunity 2020, 53, 548–563. 10.1016/j.immuni.2020.08.001. 32857950 PMC7451196

[ref23] Veneziano R.; Moyer T. J.; Stone M. B.; Wamhoff E. C.; Read B. J.; Mukherjee S.; Shepherd T. R.; Das J.; Schief W. R.; Irvine D. J.; Bathe M. Role of nanoscale antigen organization on B-cell activation probed using DNA origami. Nature Nanotechnology 2020, 15, 716–723. 10.1038/s41565-020-0719-0. PMC7415668 32601450

[ref24] Almeida J. P. M.; Lin A. Y.; Figueroa E. R.; Foster A. E.; Drezek R. A. In vivo Gold Nanoparticle Delivery of Peptide Vaccine Induces Anti-Tumor Immune Response in Prophylactic and Therapeutic Tumor Models. Small 2015, 11, 1453–1459. 10.1002/smll.201402179. 25354691 PMC4373976

[ref25] Lee B. R.; Ko H. K.; Ryu J. H.; Ahn K. Y.; Lee Y. H.; Oh S. J.; Na J. H.; Kim T. W.; Byun Y.; Kwon I. C.; Kim K.; Lee J. Engineered Human Ferritin Nanoparticles for Direct Delivery of Tumor Antigens to Lymph Node and Cancer Immunotherapy. Sci. Rep. 2016, 6, 35182 10.1038/srep35182. 27725782 PMC5057094

[ref26] Shevtsov M.; Multhoff G. Heat Shock Protein-Peptide and HSP-Based Immunotherapies for the Treatment of Cancer. Front. Immunol. 2016, 7, 171 10.3389/fimmu.2016.00171. 27199993 PMC4850156

[ref27] Caldeira J. C.; Perrine M.; Pericle F.; Cavallo F. Virus-Like Particles as an Immunogenic Platform for Cancer Vaccines. Viruses 2020, 12, 488 10.3390/v12050488. 32349216 PMC7291217

[ref28] Neek M.; Tucker J. A.; Butkovich N.; Nelson E. L.; Wang S. W. An Antigen-Delivery Protein Nanoparticle Combined with Anti-PD-1 Checkpoint Inhibitor Has Curative Efficacy in an Aggressive Melanoma Model. Adv. Ther. 2020, 3, 2000122 10.1002/adtp.202000122. PMC8205422 34141865

[ref29] Neek M.; Kim T. I.; Wang S. W. Protein-based nanoparticles in cancer vaccine development. Nanomedicine 2019, 15, 164–174. 10.1016/j.nano.2018.09.004. 30291897 PMC6289732

[ref30] Tsai S. E.; Shameli A.; Yamanouchi J.; Clemente-Casares X.; Wang J. G.; Serra P.; Yang Y.; Medarova Z.; Moore A.; Santamaria P. Reversal of Autoimmunity by Boosting Memory-like Autoregulatory T Cells. Immunity 2010, 32, 568–580. 10.1016/j.immuni.2010.03.015. 20381385

[ref31] Yeste A.; Nadeau M.; Burns E. J.; Weiner H. L.; Quintana F. J. Nanoparticle-mediated codelivery of myelin antigen and a tolerogenic small molecule suppresses experimental autoimmune encephalomyelitis. Proc. Natl. Acad. Sci. U.S.A. 2012, 109, 11270–11275. 10.1073/pnas.1120611109. 22745170 PMC3396465

[ref32] LaMothe R. A.; Kolte P. N.; Vo T.; Ferrari J. D.; Gelsinger T. C.; Wong J.; Chan V. T.; Ahmed S.; Srinivasan A.; Deitemeyer P.; Maldonado R. A.; Kishimoto T. K. Tolerogenic Nanoparticles Induce Antigen-Specific Regulatory T Cells and Provide Therapeutic Efficacy and Transferrable Tolerance against Experimental Autoimmune Encephalomyelitis. Front. Immunol. 2018, 9, 281 10.3389/fimmu.2018.00281. 29552007 PMC5840162

[ref33] Cappellano G.; Comi C.; Chiocchetti A.; Dianzani U. Exploiting PLGA-Based Biocompatible Nanoparticles for Next-Generation Tolerogenic Vaccines against Autoimmune Disease. Int. J. Mol. Sci. 2019, 20, 204 10.3390/ijms20010204. 30626016 PMC6337481

[ref34] Ahmad T. A.; Eweida A.; Sheweita S. B-cell epitope mapping for the design of vaccines and effective diagnostics. Trials Vaccinol. 2016, 5, 71–83. 10.1016/j.trivac.2016.04.003.

[ref35] Irving M. B.; Craig L.; Menendez A.; Gangadhar B. P.; Montero M.; van Houten N. E.; Scott J. K. Exploring peptide mimics for the production of antibodies against discontinuous protein epitopes. Mol. Immunol. 2010, 47, 1137–1148. 10.1016/j.molimm.2009.10.015. 20031219 PMC2821332

[ref36] Li W. D.; Joshi M. D.; Singhania S.; Ramsey K. H.; Murthy A. K. Peptide Vaccine: Progress and Challenges. Vaccines 2014, 2, 515–536. 10.3390/vaccines2030515. 26344743 PMC4494216

[ref37] Ross T. M.; Mahmood K.; Crevar C. J.; Schneider-Ohrum K.; Heaton P. M.; Bright R. A. A Trivalent Virus-Like Particle Vaccine Elicits Protective Immune Responses against Seasonal Influenza Strains in Mice and Ferrets. PLoS One 2009, 4, e6032 10.1371/journal.pone.0006032. 19554101 PMC2698286

[ref38] Palladini A.; Thrane S.; Janitzek C. M.; Pihl J.; Clemmensen S. B.; de Jongh W. A.; Clausen T. M.; Nicoletti G.; Landuzzi L.; Penichet M. L.; Balboni T.; Ianzano M. L.; Giusti V.; Theander T. G.; Nielsen M. A.; Salanti A.; Lollini P. L.; Nanni P.; Sander A. F. Virus-like particle display of HER2 induces potent anti-cancer responses. Oncoimmunology 2018, 7, e1408749 10.1080/2162402X.2017.1408749. 29399414 PMC5790387

[ref39] Allen J. D.; Jang H.; DiNapoli J.; Kleanthous H.; Ross T. M. Elicitation of Protective Antibodies against 20 Years of Future H3N2 Cocirculating Influenza Virus Variants in Ferrets Preimmune to Historical H3N2 Influenza Viruses. J. Virol. 2019, 93, e00946-18 10.1128/JVI.00946-18. PMC6340030 30429350

[ref40] Serradell M. C.; Rupil L. L.; Martino R. A.; Prucca C. G.; Carranza P. G.; Saura A.; Fernandez E. A.; Gargantini P. R.; Tenaglia A. H.; Petiti J. P.; Tonelli R. R.; Reinoso-Vizcaino N.; Echenique J.; Berod L.; Piaggio E.; Bellier B.; Sparwasser T.; Klatzmann D.; Lujan H. D. Efficient oral vaccination by bioengineering virus-like particles with protozoan surface proteins. Nat. Commun. 2019, 10, 361 10.1038/s41467-018-08265-9. 30664644 PMC6341118

[ref41] Butkovich N.; Li E. Y.; Ramirez A.; Burkhardt A. M.; Wang S. W. Advancements in protein nanoparticle vaccine platforms to combat infectious disease. Wiley Interdiscip. Rev.: Nanomed. Nanobiotechnol. 2021, 13, e1681 10.1002/wnan.1681. 33164326 PMC8052270

[ref42] Challener C. A. Fusion Proteins Pose Manufacturability Challenges. Biopharm. Int. 2017, 30, 30.

[ref43] Yang H. Q.; Liu L.; Xu F. The promises and challenges of fusion constructs in protein biochemistry and enzymology. Appl. Microbiol. Biotechnol. 2016, 100, 8273–8281. 10.1007/s00253-016-7795-y. 27541749

[ref44] Scaria P. V.; Chen B.; Rowe C. G.; Jones D. S.; Barnafo E.; Fischer E. R.; Anderson C.; MacDonald N. J.; Lambert L.; Rausch K. M.; Narum D. L.; Duffy P. E. Protein-protein conjugate nanoparticles for malaria antigen delivery and enhanced immunogenicity. PLoS One 2017, 12, e0190312 10.1371/journal.pone.0190312. 29281708 PMC5744994

[ref45] Ma W. W.; Saccardo A.; Roccatano D.; Aboagye-Mensah D.; Alkaseem M.; Jewkes M.; Di Nezza F.; Baron M.; Soloviev M.; Ferrari E. Modular assembly of proteins on nanoparticles. Nat. Commun. 2018, 9, 1489 10.1038/s41467-018-03931-4. 29662234 PMC5902510

[ref46] Lu L. T.; Duong V. T.; Shalash A. O.; Skwarczynski M.; Toth I. Chemical Conjugation Strategies for the Development of Protein-Based Subunit Nanovaccines. Vaccines 2021, 9, 563 10.3390/vaccines9060563. 34071482 PMC8228360

[ref47] Wadhwa S.; Jain A.; Woodward J. G.; Mumper R. J. Lipid nanocapsule as vaccine carriers for his-tagged proteins: Evaluation of antigen-specific immune responses to HIV I His-Gag p41 and systemic inflammatory responses. Eur. J. Pharm. Biopharm. 2012, 80, 315–322. 10.1016/j.ejpb.2011.10.016. 22068049 PMC3273636

[ref48] Chiba S.; Frey S. J.; Halfmann P. J.; Kuroda M.; Maemura T.; Yang J. E.; Wright E. R.; Kawaoka Y.; Kane R. S. Multivalent nanoparticle-based vaccines protect hamsters against SARS-CoV-2 after a single immunization. Commun. Biol. 2021, 4, 597 10.1038/s42003-021-02128-8. 34011948 PMC8134492

[ref49] Porath J.; Carlsson J.; Olsson I.; Belfrage G. Metal chelate affinity chromatography, a new approach to protein fractionation. Nature 1975, 258, 598–599. 10.1038/258598a0. 1678

[ref50] Hochuli E.; Dobeli H.; Schacher A. New metal chelate adsorbent selective for proteins and peptides containing neighboring histidine-residues. J. Chromatogr. 1987, 411, 177–184. 10.1016/S0021-9673(00)93969-4. 3443622

[ref51] Crowe J.; Döbeli H.; Gentz R.; Hochuli E.; Stüber D.; Henco K. 6xHis-Ni-NTA chromatography as a superior technique in recombinant protein expression/purification. Methods Mol. Biol. 1994, 31, 371–387. 7921034 10.1385/0-89603-258-2:371

[ref52] Soh N. Selective chemical labeling of proteins with small fluorescent molecules based on metal-chelation methodology. Sensors 2008, 8, 1004–1024. 10.3390/s8021004. 27879749 PMC3927527

[ref53] Lata S.; Reichel A.; Brock R.; Tampe R.; Piehler J. High-affinity adaptors for switchable recognition of histidine-tagged proteins. J. Am. Chem. Soc. 2005, 127, 10205–10215. 10.1021/ja050690c. 16028931

[ref54] Gatterdam K.; Joest E. F.; Gatterdam V.; Tampe R. The Scaffold Design of Trivalent Chelator Heads Dictates Affinity and Stability for Labeling His-tagged Proteins in vitro and in Cells. Angew. Chem., Int. Ed. 2018, 57, 12395–12399. 10.1002/anie.201802746. 29845721

[ref55] Irvine D. J.; Read B. J. Shaping humoral immunity to vaccines through antigen-displaying nanoparticles. Curr. Opin. Immunol. 2020, 65, 1–6. 10.1016/j.coi.2020.01.007. 32200132 PMC7501207

[ref56] Izard T.; Aelig varsson A.; Allen M. D.; Westphal A. H.; Perham R. N.; de Kok A.; Hol W. G. J. Principles of quasi-equivalence and Euclidean geometry govern the assembly of cubic and dodecahedral cores of pyruvate dehydrogenase complexes. Proc. Natl. Acad. Sci. U.S.A. 1999, 96, 1240–1245. 10.1073/pnas.96.4.1240. 9990008 PMC15447

[ref57] Dalmau M.; Lim S.; Chen H. C.; Ruiz C.; Wang S.-W. Thermostability and molecular encapsulation within an engineered caged protein scaffold. Biotechnol. Bioeng. 2008, 101, 654–664. 10.1002/bit.21988. 18814295

[ref58] Ren D. M.; Kratz F.; Wang S. W. Engineered drug-protein nanoparticle complexes for folate receptor targeting. Biochem. Eng. J. 2014, 89, 33–41. 10.1016/j.bej.2013.09.008. 25018664 PMC4090709

[ref59] Ren D. M.; Kratz F.; Wang S. W. Protein Nanocapsules Containing Doxorubicin as a pH-Responsive Delivery System. Small 2011, 7, 1051–1060. 10.1002/smll.201002242. 21456086 PMC3118673

[ref60] Molino N. M.; Neek M.; Tucker J. A.; Nelson E. L.; Wang S.-W. Display of DNA on Nanoparticles for Targeting Antigen Presenting Cells. ACS Biomater. Sci. Eng. 2017, 3, 496–501. 10.1021/acsbiomaterials.7b00148. 28989957 PMC5630166

[ref61] Molino N. M.; Anderson A. K. L.; Nelson E. L.; Wang S. W. Biomimetic Protein Nanoparticles Facilitate Enhanced Dendritic Cell Activation and Cross-Presentation. ACS Nano 2013, 7, 9743–9752. 10.1021/nn403085w. 24090491 PMC3893022

[ref62] Molino N. M.; Neek M.; Tucker J. A.; Nelson E. L.; Wang S. W. Viral-mimicking protein nanoparticle vaccine for eliciting anti-tumor responses. Biomaterials 2016, 86, 83–91. 10.1016/j.biomaterials.2016.01.056. 26894870 PMC4775383

[ref63] Neek M.; Tucker J. A.; Kim T. I.; Molino N. M.; Nelson E. L.; Wang S.-W. Co-delivery of human cancer-testis antigens with adjuvant in protein nanoparticles induces higher cell-mediated immune responses. Biomaterials 2018, 156, 194–203. 10.1016/j.biomaterials.2017.11.022. 29202325 PMC5783197

[ref64] Zhang Y. N.; Lazarovits J.; Poon W.; Ouyang B.; Nguyen L. N. M.; Kingston B. R.; Chan W. C. W. Nanoparticle Size Influences Antigen Retention and Presentation in Lymph Node Follicles for Humoral Immunity. Nano Lett. 2019, 19, 7226–7235. 10.1021/acs.nanolett.9b02834. 31508968

[ref65] World-Health-Organization. Global Influenza Programme—Burden of Disease; World-Health-Organization, 2022.

[ref66] Houser K.; Subbarao K. Influenza Vaccines: Challenges and Solutions. Cell Host Microbe 2015, 17, 295–300. 10.1016/j.chom.2015.02.012. 25766291 PMC4362519

[ref67] Wei C. J.; Crank M. C.; Shiver J.; Graham B. S.; Mascola J. R.; Nabel G. J. Next-generation influenza vaccines: opportunities and challenges. Nat. Rev. Drug Discovery 2020, 19, 239–252. 10.1038/s41573-019-0056-x. 32060419 PMC7223957

[ref68] Watson D. S.; Platt V. M.; Cao L. M.; Venditto V. J.; Szoka F. C. Antibody Response to Polyhistidine-Tagged Peptide and Protein Antigens Attached to Liposomes via Lipid-Linked Nitrilotriacetic Acid in Mice. Clin. Vaccine Immunol. 2011, 18, 289–297. 10.1128/CVI.00425-10. 21159923 PMC3067350

[ref69] Platt V.; Huang Z. H.; Cao L. M.; Tiffany M.; Riviere K.; Szoka F. C. Influence of Multivalent Nitrilotriacetic Acid Lipid-Ligand Affinity on the Circulation Half-Life in Mice of a Liposome-Attached His(6)-Protein. Bioconjugate Chem. 2010, 21, 892–902. 10.1021/bc900448f. PMC2874083 20384362

[ref70] Li Y. M.; Li A. C.; Xu Q. B. Intracellular Delivery of His-Tagged Genome-Editing Proteins Enabled by Nitrilotriacetic Acid-Containing Lipidoid Nanoparticles. Adv. Healthcare Mater. 2019, 8, 1800996 10.1002/adhm.201800996. PMC6474682 30565897

[ref71] van Broekhoven C. L.; Altin J. G. The novel chelator lipid 3(nitrilotriacetic acid)-ditetradecylamine (NTA(3)-DTDA) promotes stable binding of His-tagged proteins to liposomal membranes: Potent anti-tumor responses induced by simultaneously targeting antigen, cytokine and costimulatory signals to T cells. Biochim. Biophys. Acta, Biomembr. 2005, 1716, 104–116. 10.1016/j.bbamem.2005.09.003 16225839

[ref72] Milder F. J.; Jongeneelen M.; Ritschel T.; Bouchier P.; Bisschop I. J. M.; de Man M.; Veldman D.; Le L.; Kaufmann B.; Bakkers M. J. G.; Juraszek J.; Brandenburg B.; Langedijk J. P. M. Universal stabilization of the influenza hemagglutinin by structure-based redesign of the pH switch regions. Proc. Natl. Acad. Sci. U.S.A. 2022, 119, e2115379119 10.1073/pnas.2115379119. 35131851 PMC8833195

[ref73] McMillan C. L. D.; Cheung S. T. M.; Modhiran N.; Barnes J.; Amarilla A. A.; Bielefeldt-Ohmann H.; Lee L. Y. Y.; Guilfoyle K.; van Amerongen G.; Stittelaar K.; Jakon V.; Lebas C.; Reading P.; Short K. R.; Young P. R.; Watterson D.; Chappell K. J. Development of molecular clamp stabilized hemagglutinin vaccines for Influenza A viruses. npj Vaccines 2021, 6, 135 10.1038/s41541-021-00395-4. 34750396 PMC8575991

[ref74] Macioła A. K.; Pietrzak M. A.; Kosson P.; Czarnocki-Cieciura M.; Smietanka K.; Minta Z.; Kopera E. The Length of N-Glycans of Recombinant H5N1 Hemagglutinin Influences the Oligomerization and Immunogenicity of Vaccine Antigen. Front. Immunol. 2017, 8, 444 10.3389/fimmu.2017.00444. 28473830 PMC5397403

[ref75] Pietrzak M.; Maciola A.; Zdanowski K.; Protas-Klukowska A. M.; Olszewska M.; Smietanka K.; Minta Z.; Szewczyk B.; Kopera E. An avian influenza H5N1 virus vaccine candidate based on the extracellular domain produced in yeast system as subviral particles protects chickens from lethal challenge. Antiviral Res. 2016, 133, 242–249. 10.1016/j.antiviral.2016.08.001. 27498036

[ref76] Aartse A.; Eggink D.; Claireaux M.; van Leeuwen S.; Mooij P.; Bogers W. M.; Sanders R. W.; Koopman G.; van Gils M. J. Influenza A Virus Hemagglutinin Trimer, Head and Stem Proteins Identify and Quantify Different Hemagglutinin-Specific B Cell Subsets in Humans. Vaccines 2021, 9, 717 10.3390/vaccines9070717. 34358138 PMC8310015

[ref77] Copeland C. S.; Doms R. W.; Bolzau E. M.; Webster R. G.; Helenius A. Assembly of influenza hemagglutinin trimers and its role in intracellular-transport. J. Cell Biol. 1986, 103, 1179–1191. 10.1083/jcb.103.4.1179. 2429970 PMC2114319

[ref78] Hernandez-Davies J. E.; Dollinger E. P.; Pone E. J.; Felgner J.; Liang L.; Strohmeier S.; Jan S.; Albin T. J.; Jain A.; Nakajima R.; Jasinskas A.; Krammer F.; Esser-Kahn A.; Felgner P. L.; Nie Q.; Davies D. H. Magnitude and breadth of antibody cross-reactivity induced by recombinant influenza hemagglutinin trimer vaccine is enhanced by combination adjuvants. Sci. Rep. 2022, 12, 9198 10.1038/s41598-022-12727-y. 35654904 PMC9163070

[ref79] Reddy S. T.; van der Vlies A. J.; Simeoni E.; Angeli V.; Randolph G. J.; O’Neill C. P.; Lee L. K.; Swartz M. A.; Hubbell J. A. Exploiting lymphatic transport and complement activation in nanoparticle vaccines. Nat. Biotechnol. 2007, 25, 1159–1164. 10.1038/nbt1332. 17873867

[ref80] Manolova V.; Flace A.; Bauer M.; Schwarz K.; Saudan P.; Bachmann M. F. Nanoparticles target distinct dendritic cell populations according to their size. Eur. J. Immunol. 2008, 38, 1404–1413. 10.1002/eji.200737984. 18389478

[ref81] Vos Q.; Lees A.; Wu Z. Q.; Snapper C. M.; Mond J. J. B-cell activation by T-cell-independent type 2 antigens as an integral part of the humoral immune response to pathogenic microorganisms. Immunol. Rev. 2000, 176, 154–170. 10.1034/j.1600-065X.2000.00607.x. 11043775

[ref82] Turner J. S.; Ke F.; Grigorova I. L. B Cell Receptor Crosslinking Augments Germinal Center B Cell Selection when T Cell Help Is Limiting. Cell Rep. 2018, 25, 1395–1403. 10.1016/j.celrep.2018.10.042. 30403996 PMC6289055

[ref83] Viola A.; Lanzavecchia A. T cell activation determined by T cell receptor number and tunable thresholds. Science 1996, 273, 104–106. 10.1126/science.273.5271.104. 8658175

[ref84] Kimachi K.; Croft M.; Grey H. M. The minimal number of antigen-major histocompatibility complex class II complexes required for activation of naive and primed T cells. Eur. J. Immunol. 1997, 27, 3310–3317. 10.1002/eji.1830271230. 9464819

[ref85] Mountford A. P.; Fisher A.; Wilson R. A. The profile of igg1 and igg2a antibody-responses in mice exposed to schistosoma-mansoni. Parasite Immunol. 1994, 16, 521–527. 10.1111/j.1365-3024.1994.tb00306.x. 7870462

[ref86] Visciano M. L.; Tagliamonte M.; Tornesello M. L.; Buonaguro F. M.; Buonaguro L. Effects of adjuvants on IgG subclasses elicited by virus-like Particles. J. Transl. Med. 2012, 10, 8 10.1186/1479-5876-10-4. 22221900 PMC3311067

[ref87] Rostamian M.; Sohrabi S.; Kavosifard H.; Niknam H. M. Lower levels of IgG1 in comparison with IgG2a are associated with protective immunity against Leishmania tropica infection in BALB/c mice. J. Microbiol., Immunol. Infect. 2017, 50, 160–166. 10.1016/j.jmii.2015.05.007. 26066544

[ref88] Shi S. T.; Zhu H. R.; Xia X. Y.; Liang Z. H.; Ma X. H.; Sun B. B. Vaccine adjuvants: Understanding the structure and mechanism of adjuvanticity. Vaccine 2019, 37, 3167–3178. 10.1016/j.vaccine.2019.04.055. 31047671

[ref89] Schotsaert M.; Saelens X.; Leroux-Roels G. Influenza vaccines: T-cell responses deserve more attention. Expert Rev. Vaccines 2012, 11, 949–962. 10.1586/erv.12.71. 23002976

[ref90] Sridhar S.; Begom S.; Bermingham A.; Hoschler K.; Adamson W.; Carman W.; Bean T.; Barclay W.; Deeks J. J.; Lalvani A. Cellular immune correlates of protection against symptomatic pandemic influenza. Nat. Med. 2013, 19, 1305–1312. 10.1038/nm.3350. 24056771

[ref91] Braciale T. Immunologic recognition of influenza virus-infected cells. I. Generation of a virus-strain specific and a cross-reactive subpopulation of cytotoxic T cells in the response to type A influenza viruses of different subtypes. Cell. Immunol. 1977, 33, 423–436. 10.1016/0008-8749(77)90170-8. 303150

[ref92] Lu L. Y.; Askonas B. A. Cross-reactivity for different type-a influenza-viruses of a cloned t-killer cell-line. Nature 1980, 288, 164–165. 10.1038/288164a0. 6968871

[ref93] Yap K. L.; Ada G. L.; McKenzie I. F. C. Transfer of specific cytotoxic t-lymphocytes protects mice inoculated with influenza-virus. Nature 1978, 273, 238–239. 10.1038/273238a0. 306072

[ref94] Bender B. S.; Croghan T.; Zhang L. P.; Small P. A. Transgenic mice lacking class-I major histocompatibility complex-restricted t-cells have delayed viral clearance and increased mortality after influenza-virus challenge. J. Exp. Med. 1992, 175, 1143–1145. 10.1084/jem.175.4.1143. 1552285 PMC2119177

[ref95] Miyauchi K.; Sugimoto-Ishige A.; Harada Y.; Adachi Y.; Usami Y.; Kaji T.; Inoue K.; Hasegawa H.; Watanabe T.; Hijikata A.; Fukuyama S.; Maemura T.; Okada-Hatakeyama M.; Ohara O.; Kawaoka Y.; Takahashi Y.; Takemori T.; Kubo M. Protective neutralizing influenza antibody response in the absence of T follicular helper cells. Nat. Immunol. 2016, 17, 1447–1458. 10.1038/ni.3563. 27798619

[ref96] Gu X.; Li P.; Liu H.; Li N.; Li S.; Sakuma T. The effect of influenza virus A on th1/th2 balance and alveolar fluid clearance in pregnant rats. Exp. Lung Res. 2011, 37, 445–451. 10.3109/01902148.2011.587136. 21777148

[ref97] Drake J. W. Rates of spontaneous mutation among rna viruses. Proc. Natl. Acad. Sci. U.S.A. 1993, 90, 4171–4175. 10.1073/pnas.90.9.4171. 8387212 PMC46468

[ref98] Skehel J. J.; Waterfield M. D. Studies on primary structure of influenza-virus hemagglutinin. Proc. Natl. Acad. Sci. U.S.A. 1975, 72, 93–97. 10.1073/pnas.72.1.93. 1054518 PMC432247

[ref99] Nobusawa E.; Aoyama T.; Kato H.; Suzuki Y.; Tateno Y.; Nakajima K. Comparison of complete amino-acid-sequences and receptor-binding properties among 13 serotypes of hemagglutinins of influenza a-viruses. Virology 1991, 182, 475–485. 10.1016/0042-6822(91)90588-3. 2024485

[ref100] Kirkpatrick E.; Qiu X. T.; Wilson P. C.; Bahl J.; Krammer F. The influenza virus hemagglutinin head evolves faster than the stalk domain. Sci. Rep. 2018, 8, 10432 10.1038/s41598-018-28706-1. 29992986 PMC6041311

[ref101] Lee J. M.; Huddleston J.; Doud M. B.; Hooper K. A.; Wu N. C.; Bedford T.; Bloom J. D. Deep mutational scanning of hemagglutinin helps predict evolutionary fates of human H3N2 influenza variants. Proc. Natl. Acad. Sci. U.S.A. 2018, 115, EB276–EB285. 10.1073/pnas.1806133115. PMC6126756 30104379

[ref102] Hernandez-Davies J. E.; Felgner J.; Strohmeier S.; Pone E. J.; Jain A.; Jan S.; Nakajima R.; Jasinskas A.; Strahsburger E.; Krammer F.; Felgner P. L.; Davies D. H. Administration of Multivalent Influenza Virus Recombinant Hemagglutinin Vaccine in Combination-Adjuvant Elicits Broad Reactivity Beyond the Vaccine Components. Front. Immunol. 2021, 12, 18 10.3389/fimmu.2021.692151. PMC8318558 34335601

[ref103] Zost S. J.; Wu N. C.; Hensley S. E.; Wilson I. A. Immunodominance and Antigenic Variation of Influenza Virus Hemagglutinin: Implications for Design of Universal Vaccine Immunogens. J. Infect. Dis. 2019, 219, S38–S45. 10.1093/infdis/jiy696. 30535315 PMC6452323

[ref104] Fukuyama H.; Shinnakasu R.; Kurosaki T. Influenza vaccination strategies targeting the hemagglutinin stem region. Immunol. Rev. 2020, 296, 132–141. 10.1111/imr.12887. 32542739 PMC7323124

[ref105] Wu N. C.; Wilson I. A. Structural Biology of Influenza Hemagglutinin: An Amaranthine Adventure. Viruses 2020, 12, 1053 10.3390/v12091053. 32971825 PMC7551194

[ref106] Wan X. F. Lessons from Emergence of A/Goose/Guangdong/1996-Like H5N1 Highly Pathogenic Avian Influenza Viruses and Recent Influenza Surveillance Efforts in Southern China. Zoonoses Public Health 2012, 59, 32–42. 10.1111/j.1863-2378.2012.01497.x. 22958248 PMC4119829

[ref107] Molino N. M.; Bilotkach K.; Fraser D. A.; Ren D.; Wang S.-W. Complement Activation and Cell Uptake Responses Toward Polymer-Functionalized Protein Nanocapsules. Biomacromolecules 2012, 13, 974–981. 10.1021/bm300083e. 22416762 PMC3322319

[ref108] Gasteiger E.; Hoogland C.; Gattiker A.; Duvaud S.; Wilkins M. R.; Appel R. D.; Bairoch A. Protein Identification and Analysis Tools on the ExPASy Server, in The Proteomics Protocols Handbook. In Springer Protocols Handbooks; Humana Press, 2005.

[ref109] Goddard T. D.; Huang C. C.; Meng E. C.; Pettersen E. F.; Couch G. S.; Morris J. H.; Ferrin T. E. UCSF ChimeraX: Meeting modern challenges in visualization and analysis. Protein Sci. 2018, 27, 14–25. 10.1002/pro.3235. 28710774 PMC5734306

[ref110] Pettersen E. F.; Goddard T. D.; Huang C. R. C.; Meng E. E. C.; Couch G. S.; Croll T. I.; Morris J. H.; Ferrin T. E. UCSF ChimeraX: Structure visualization for researchers, educators, and developers. Protein Sci. 2021, 30, 70–82. 10.1002/pro.3943. 32881101 PMC7737788

[ref111] Nakajima R.; Supnet M.; Jasinskas A.; Jain A.; Taghavian O.; Obiero J.; Milton D. K.; Chen W. H.; Grantham M.; Webby R.; Krammer F.; Carter D.; Felgner P. L.; Davies D. H. Protein Microarray Analysis of the Specificity and Cross-Reactivity of Influenza Virus Hemagglutinin-Specific Antibodies. mSphere 2018, 3, 2530 10.1128/mSphere.00592-18. PMC6291623 30541779

