# Supplementary material for: Protein Nanoparticle-Mediated Delivery of Recombinant Influenza Hemagglutinin Enhances Immunogenicity and Breadth of the Antibody Response
Source: ACS Infect Dis. 2023 Jan 6;9(2):239–52. doi: 10.1021/acsinfecdis.2c00362 (PMC9926493; doi:10.1021/acsinfecdis.2c00362)
Supplement: Supplementary file 1 — id2c00362_si_001.pdf [file id2c00362_si_001.pdf]

Supporting Information

Protein Nanoparticle-Mediated Delivery of Recombinant Influenza Hemagglutinin Enhances Immunogenicity and Breadth of the Antibody Response

Alexander J. Badten[1,2,§], Aaron Ramirez[1,§], Jenny E. Hernandez-Davies[2], Tyler J. Albin[3], Aarti Jain[2], Rie Nakajima[2], Jiin Felgner[2], D. Huw Davies[2,6*], Szu-Wen Wang[1,4,5,6,*]

[1] Department of Chemical and Biomolecular Engineering
[2] Vaccine Research and Development Center, Department of Physiology and Biophysics
[3] Department of Chemistry
[4] Department of Biomedical Engineering
[5] Chao Family Comprehensive Cancer Center
[6] Institute for Immunology
University of California
Irvine, CA 92697 USA

[§] These authors contributed equally to this manuscript and are co-first authors.

*Co-corresponding authors:
Szu-Wen Wang, wangsw@uci.edu
D. Huw Davies, ddavies@uci.edu

Supporting information: Methods

Synthetic Methods:

(N,N) di-*t*-butyl acetate-L-Glu(Bzl)-OtBu (*2*). Reaction was performed similarly as previously reported [1]. t-butyl bromoacetate (3.558 mL, 4.681 g, 24 mmol) and DIPEA (5.3 mL, 3.877 g, 30 mmol) were added to a solution of H-L-Glu(Bzl)-OtBu hydrochloride (***1***) (1.979 g, 6 mmol) in DMF (50 mL). The reaction was purged with nitrogen gas and heated to 55 °C for 12 h with continuous stirring. The solvent was removed by rotary evaporation at 60 °C. Ethyl acetate (20 mL) was added to the reaction residue to obtain a slurry and was filtered and the liquid collected. The precipitate was washed 3X with hexane/ethyl acetate (3:1, 3X 40 mL) and the liquid collected. The combined filtrate and washes were concentrated by rotary evaporation. The product was purified by flash chromatography using hexane/ethyl acetate. Column Conditions: mobile phase A: hexanes, mobile phase B: ethyl acetate. Gradient: 0-3 min 0% B, 3-7 min ramp to 15% B, 7-15 min 100% B. Product eluted as a peak between 9.5-12 min. The fractions containing product were pooled, concentrated by rotary evaporation, dried over high vacuum, and weighed. The product was confirmed by NMR and TLC in cyclohexane/ethyl acetate (Rf = 0.6) and was obtained in 81% yield (2.535 g, 4.866 mmol). 1H NMR (500 MHz,$CDCl_3$) δ 7.37-7.28 (m, 5H), 5.11 (s, 2H), 3.43 (s, 4H), 3.38 (dd, 1H), 2.73 – 2.56 (m, 2H), 2.04-2.87 (m, 2H), 1.45 (s, 9H), 1.43 (s, 18H). 13C NMR (126 MHz, $CDCl_3$) δ 173.6, 171.9, 170.6, 136.3, 128.6, 128.3, 128.2, 81.4, 80.8, 66.2, 64.5, 54.0, 30.7, 28.3, 28.2, 25.5.

(N,N) di-*t*-butylacetate-L-Glu-OtBu (*3*). Reaction was performed similarly as previously reported [1]. 10% Pd/C was added to a flame dried round bottom flask with a magnetic stir bar and purged with nitrogen gas. Methanol (110 mL) was added to the flask, followed by compound **1** (2.535 g) dissolved in methanol (10 mL). The solution was purged again with nitrogen gas. A hydrogen balloon was then added and the reaction ran for 6 h. The reaction solution was passed through a celite plug to remove Pd/C. The methanol was removed by rotary evaporation, transferred to a pre-weighed vial, and dried under high vacuum. The product was weighed and characterized by NMR. The product was obtained in 95% yield (1.994 g, 4.621 mmol). 1H NMR (500 MHz,$CDCl_3$) δ 3.43 (s, 4H), 3.36 (dd, 1H), 2.63 (m, 2H), 1.95 (m, 2H), 1.45 (s, 9H), 1.43 (s, 18H). 13C NMR (126 MHz, $CDCl_3$) δ 177.6, 171.6, 170.7, 81.7, 81.27, 64.7, 54.1, 31.0, 28.3, 28.2, 25.5.

t-butyl protected tris-NTA-NH (*4*). Reaction was performed similarly as previously reported [1]. Compound ***2*** (3.495 g, 8.100 mmol) and HBTU (3.351 g, 8.837 mmol) were added to a flame dried round bottom flask and dissolved in DCM/DMF (1:1, 100 mL). DIPEA (10 mL) was added to the reaction mixture and the solution stirred for 5 min. Tetraazacyclodecane (0.487 g, 2.430 mmol) was added to the reaction mixture and the reaction stirred for 18 h. The solvent was removed by rotary evaporation at 50 °C. The reaction residue was dissolved in hexanes/ethyl acetate and purified by flash chromatography. Column conditions: mobile phase A: hexanes, mobile phase B: ethyl acetate. Gradient: 0-5 min 0% B, 5-15 min ramp to 100% B, 15-25 min 100% B. Product eluted as a broad peak at 15-22 min. The fractions containing product were combined, the solvent removed by rotary evaporation, and the product dried under high vacuum which was obtained in 53% yield (1.861 g, 1.292 mmol).

t-butyl protected-tris-NTA-mal. Maleimido-propionic acid (27 mg, 0.16 mmol) and HBTU (61 mg, 0.16 mmol) was dissolved in DMF (9.5 mL) and DIPEA (0.5 mL). After five minutes, t-butyl protected-tris-NTA-NH (151 mg, 0.11 mmol) was added and the reaction was stirred overnight. The solvent was removed by rotary evaporation and the product purified by flash chromatography. Column conditions: 40 g silica gel column, Mobile phase A: hexanes, mobile phase B: ethyl acetate. Gradient: percent mobile phase B: 0-4 min 0% B, 4-12 min 100% B ramp, 12-20 min 100% B. Product eluted at 13 min. The fractions containing product were collected, the solvent removed by rotary evaporation, and dried over high vacuum. The product was recovered and analyzed by ESI-MS (110 mg, 66% yield). ESI was performed on a Waters LCT ESI MS with flow injection at 0.1mL/min in 100% MeOH. Predicted $[M+Na]^+$: 1613.9 m/z Observed $[M+Na]^+$: 1612.9 m/z.

mal-tNTA (*5*). t-butyl protected tris-NTA-mal (109 mg, 0.07 mmol) was dissolved in 95% TFA in water (5 mL) and stirred for 2 h. The TFA was removed by rotary evaporation and the residue added to 40 mL of cold diethyl ether to precipitate the product. The mixture was centrifuged to recover the product pellet. The pellet was dissolved in 50% water/ACN, sterile filtered through a nylon 0.22 μm filter, and lyophilized. The product (*5*) was weighed and analyzed by LC-MS in water/acetonitrile with 0.1% formic acid (53 mg, 71% yield). LC-MS was performed on a Waters LC-MS with QDA detector with Hclass UPLC with a water/acetonitrile 0.1% formic acid solvent system. Predicted [M+H]+: 1087.4 m/z Observed [M+H]+: 1087.8 m/z.

Attachment of His_6-Tagged GFP to tNTA-E2

A 10X molar excess of aqueous $NiCl_2$ was first added to the tNTA-E2 and incubated for 2 hours at room temperature on a shaker. The sample was then run through a 0.5mL 40kDa molecular weight cutoff Zeba spin desalting column according to manufacturer's instructions to remove unchelated $NiCl_2$. A 1:2.2 molar ratio (E2 monomer:GFP) of his_6-tagged GFP was added to Ni-tNTA-E2 or tNTA-E2 and incubated at room temperature on a shaker for 2 hours. Reaction solutions were then run through a packed Sephacryl S-200 SEC column via gravity separation to remove unreacted reactants. Elution profiles and loading quantifications of GFP-$(His)_6$ were determined with spectrophotometry at 280 nm and fluorescence spectrometry of GFP (Ex: 488 nm and Em: 510 nm).

Supporting information: Figures

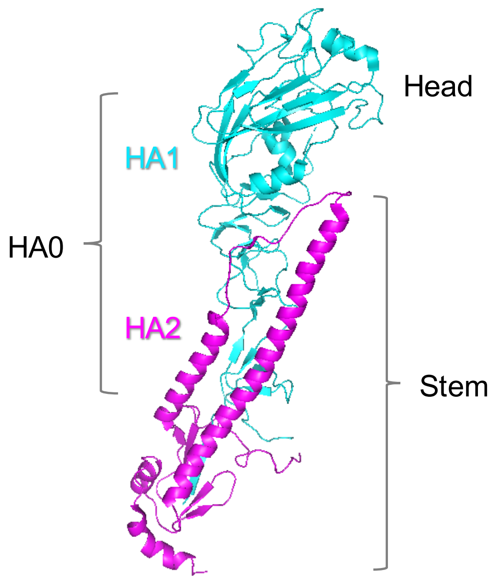

Figure SI-1. Three-dimensional representation of the HA monomer with HA1 sequence shown in cyan and HA2 sequence in magenta (PDB: 3ztn).

A

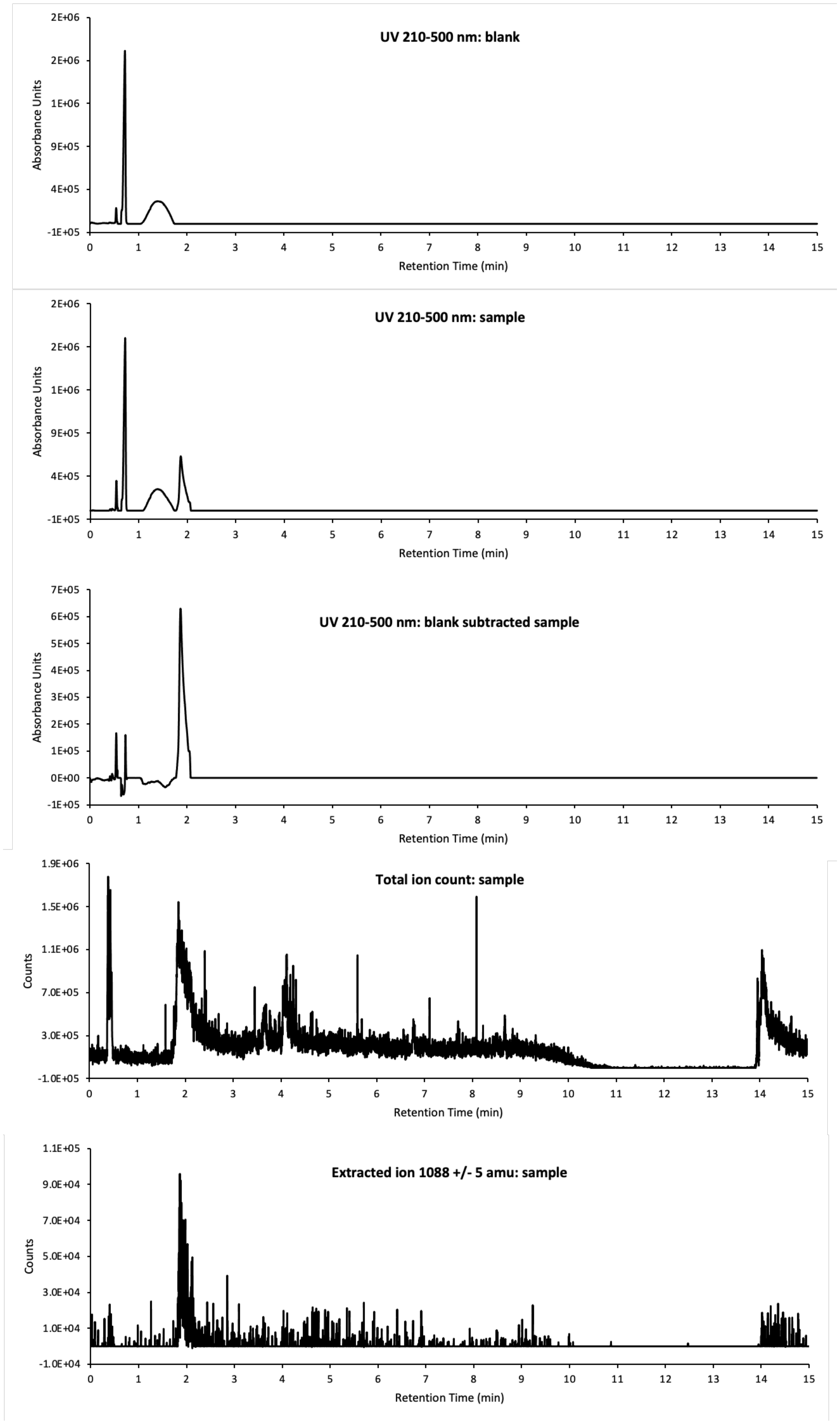
UV 210-500 nm: blank
Absorbance Units
2E+06
2E+06
1E+06
9E+05
4E+05
-1E+05
Retention Time (min)
UV 210-500 nm: sample
Absorbance Units
Retention Time (min)
UV 210-500 nm: blank subtracted sample
Absorbance Units
7E+05
6E+05
5E+05
4E+05
3E+05
2E+05
1E+05
0E+00
-1E+05
Retention Time (min)
Total ion count: sample
Counts
1.9E+06
1.5E+06
1.1E+06
7.0E+05
3.0E+05
-1.0E+05
Retention Time (min)
Extracted ion 1088 +/- 5 amu: sample
Counts
1.1E+05
9.0E+04
7.0E+04
5.0E+04
3.0E+04
1.0E+04
-1.0E+04
Retention Time (min)
0 1 2 3 4 5 6 7 8 9 10 11 12 13 14 15

B

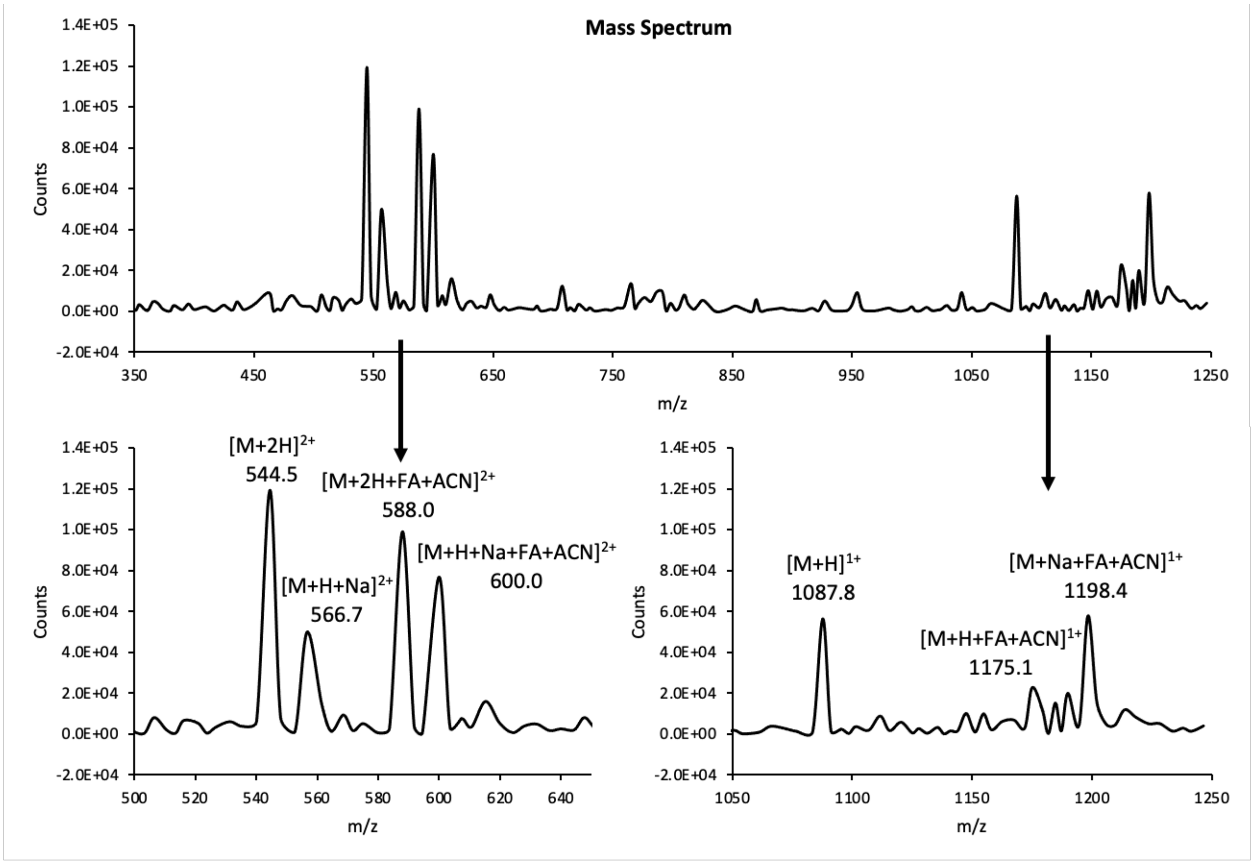

Figure SI-2. LC-MS data of maleimido tris-NTA (mal-tNTA). A. Traces show chromatograms from final product UV and ions detected. B. Mass spectrum of main peak, confirming identity of mal-tNTA.

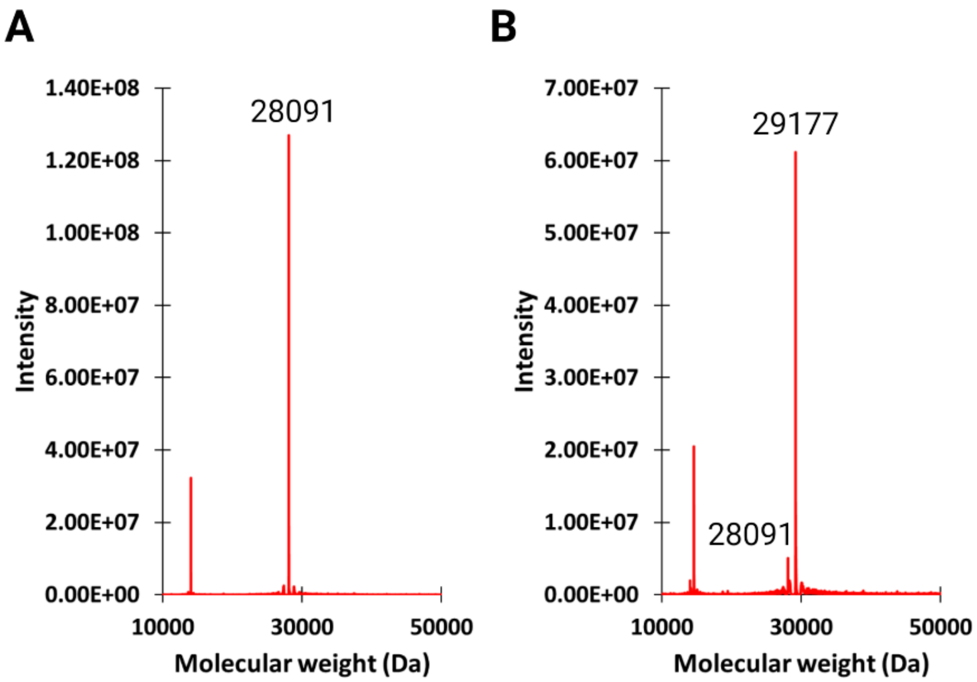

Figure SI-3. Representative mass spectrometry data for (a) E2 protein (E279C E2) and (b) E2 conjugated with mal-tNTA (tNTA-E2). Because the 60-mer nanoparticle is assembled via non-covalent intermolecular interactions, the use of relatively high-energy, non-native conditions in the ESI-MS instrument dissociates the protein nanoparticles into its monomers, yielding monomeric molecular weight values.

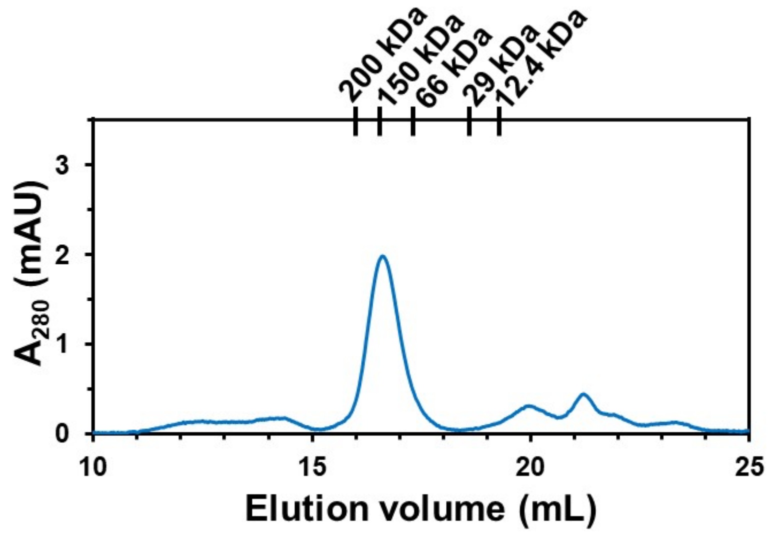

Figure SI-4: Size exclusion chromatography (SEC) of H1. H1 is shown as solid blue line. Elution volumes of molecular weight (MW) standards are marked at the top of the chromatogram. The SEC elution peak of our soluble H1 (as-received) is located between 66 and 150 kDa, and this is consistent with other studies reporting monomeric HA proteins (with trimeric forms eluting at molecular weights > 400 kDa) [2-5]. MW standards included: β-amylase (200 kDa), alcohol dehydrogenase (150 kDa), albumin bovine serum (66 kDa), carbonic anhydrase (29 kDa), and cytochrome c (12.4 kDa). MW standards were run according to manufacturer's suggestions (Sigma Aldrich Cat#: MWGF200), on a Superose™ 6 increase 10/300 GL analytical column (Cytiva). A comparison of our H1 elution profile with H1 monomer and H1 trimer elution profiles from McMillan et al.[3] supports that our H1 protein is in monomeric form. No significant A_{280} signal was observed from 0-10 mL.

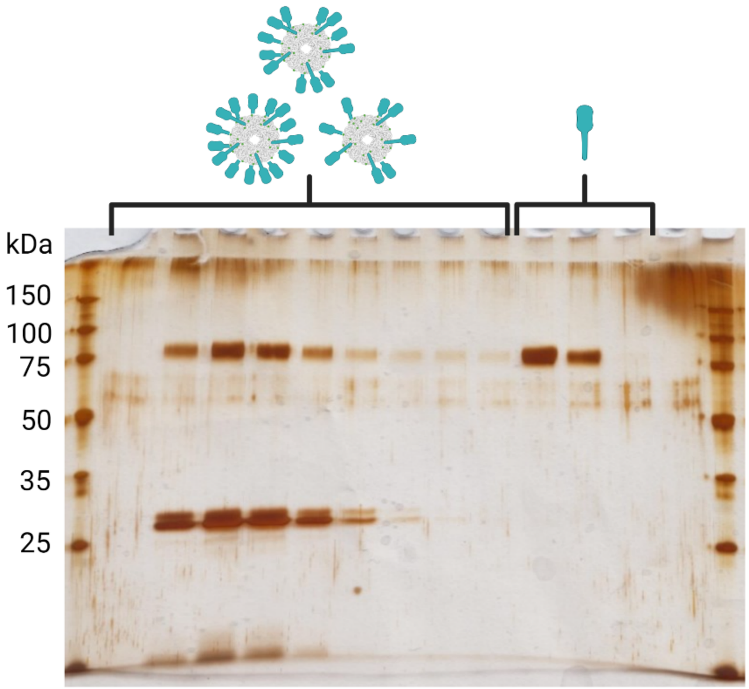

Figure SI-5. SDS-PAGE with silver stain of fractions collected from analytical SEC used to separate H1-E2 from unbound H1. Each collected fraction was analyzed with SDS-PAGE, and confirmed H1-bound E2 in the first SEC peak and unbound H1 in the second peak.

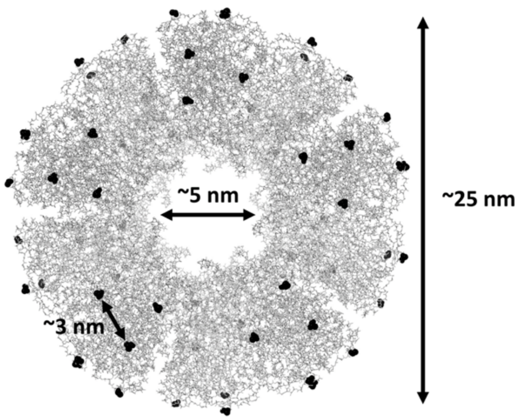

Figure SI-6. ChimeraX-generated three-dimensional structure of E2 nanoparticle (PDB: 1b5s). Black spots are located at amino acid position 279, the site of the engineered cysteine residue (to yield mutant E279C) to which the mal-tNTA for conjugating the His-tagged protein antigen is bound.

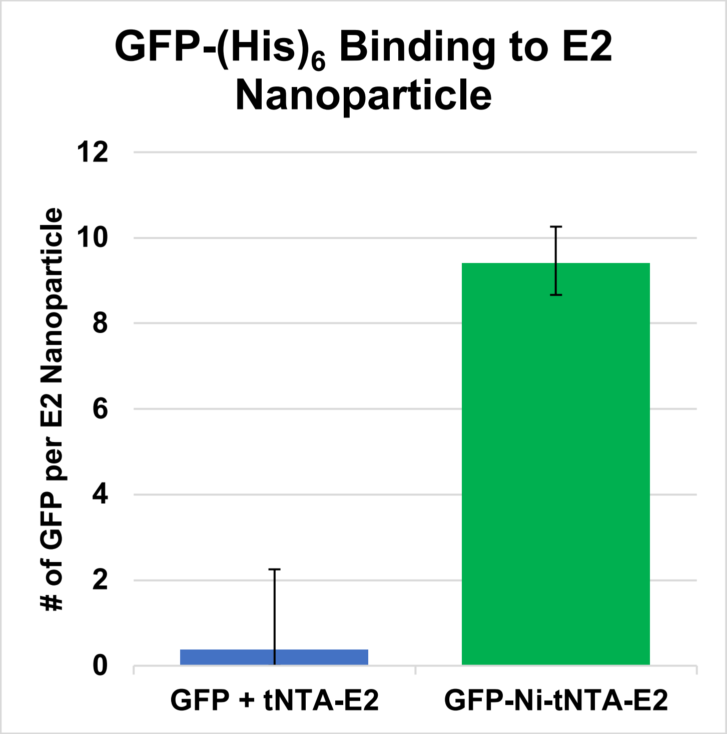

Figure SI-7. GFP attachment to E2. Bar graph of the number of GFP bound to Ni-tNTA-E2 after high affinity Ni-tNTA + $(His)_n$-protein conjugation. tNTA-E2 is not loaded with Ni, thus no binding is expected. Ni-tNTA-E2 is loaded with Ni, thus binding is expected.

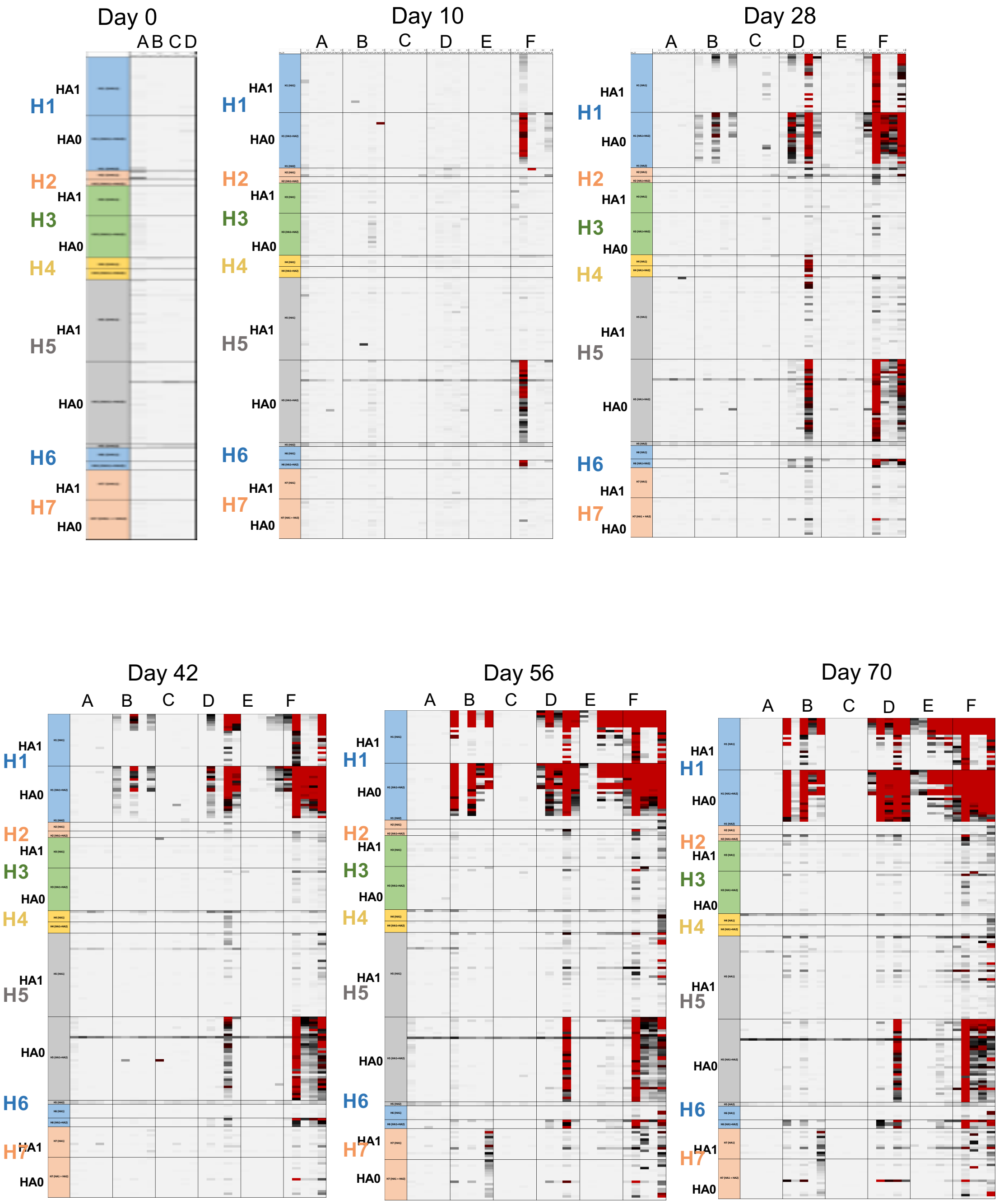
Day 0
A B C D
Day 10
Day 28
Day 42
Day 56
Day 70
A
B
C
D
E
F
H1
H2
H3
H4
H5
H6
H7
HA1
HA0

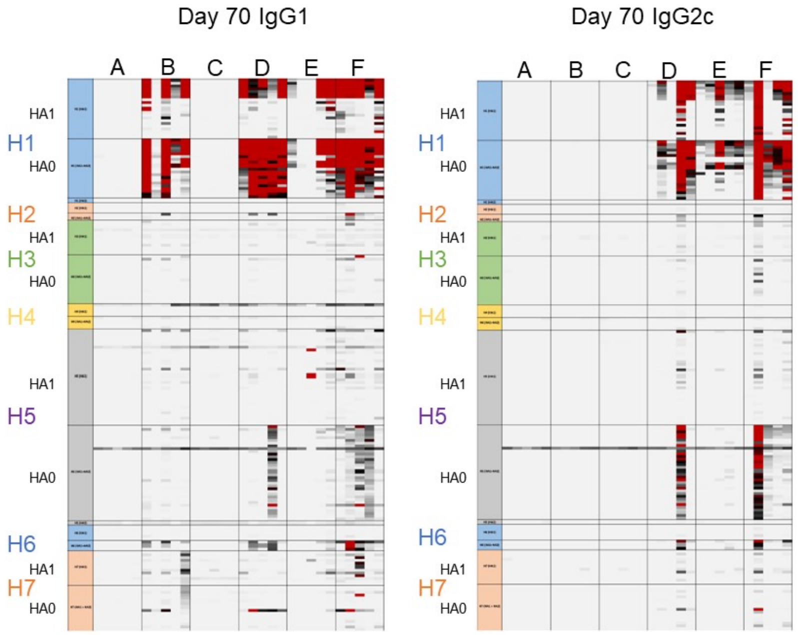

Figure SI-8: Antibody arrays from sera of immunized mice. Six groups of 5 B6 mice (Groups A to F) were administered different formulations on d0 and boosted on d14 and d49. IgG, IgG1, and IgG2c signal intensities were determined at different time points post-prime against HA variants displayed on protein microarrays (Signals to H1 through H7 variants are shown). Microarray data was compiled into heat map compilations with columns representing immunized mice groups and rows representing individual HA variants and their specific HA1 and HA0 domains (red = high amount of binding, white = low amount of binding). Within each HA1 and HA0 row were the individual domains of homosubtypic HA variants (i.e., 23 variants of H1 were analyzed). Group A: PBS, Group B: H1, Group C: H1 + E2, Group D: H1 + E2 + MPLA, Group E: H1-E2, Group F: H1-E2 + MPLA. Day 0 sera = naive mice.

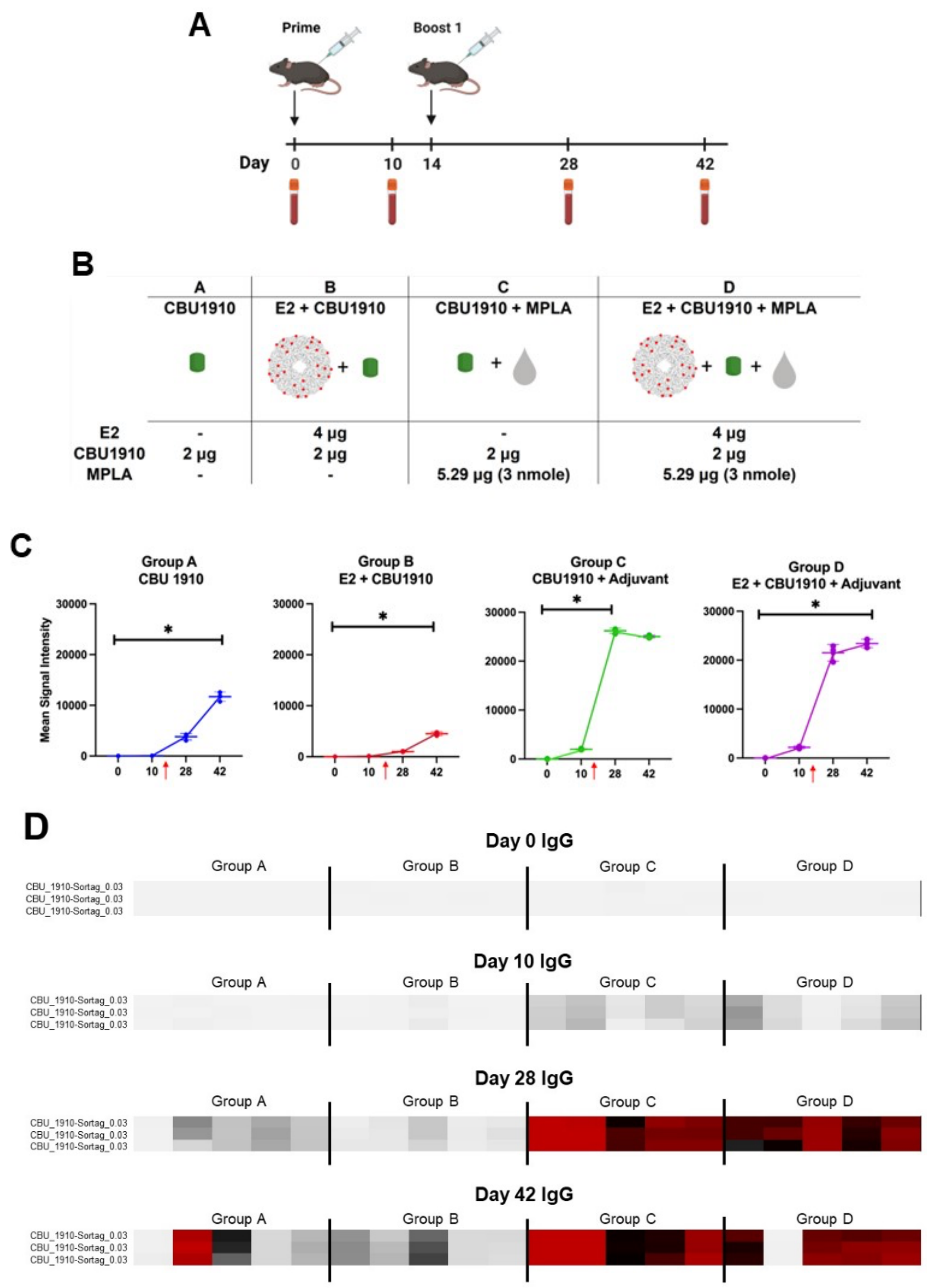

Figure SI-9: CBU1910-specific IgG profiling by protein microarray shows potential antibody suppression from co-mixing of E2 with protein target antigen, CBU1910.

A) Timeline of immunizations and plasma collection. **B)** Table summarizing the vaccine groups and dose amounts per injection. Group A) CBU1910 only; Group B) E2 nanoparticle and CBU1910 (unconjugated); Group C) CBU910 with MPLA (TLR4 agonist); Group D) E2 nanoparticle and CBU1910 (unconjugated), with MPLA (TLR4 agonist). **C)** CBU1910-specific IgG profiling by protein microarray. Four groups of 5 B6 mice (Groups A to D) were administered

different formulations as indicated and boosted on d14 (red arrows). Array data are shown as dots plots of IgG signal intensities at different time points post-prime against CBU1910 displayed on a *Coxiella burnetti* protein microarray; each dot represents a replicate (of 3) of CBU1910 (mean of 5 mice) with lines connecting the means (± SD error bars). One-way ANOVA (non-parametric) comparisons using a Kruskal-Wallis test were made between both pre- and post-boost time points: *$p<0.05$. **D)** Microarray raw data was compiled into heat map compilations with columns representing immunized mice groups and rows representing CBU1910 (red = high amount of binding, white = low amount of binding). Abbreviations: MPLA, monophosphoryl lipid A.

A

Influenza Strain/Control ID	Influenza Type	Subtype
A/Beijing/22808/2009	A	H1N1
A/Texas/05/2009	A	H1N1
A/England/195/2009	A	H1N1
A/New York/18/2009	A	H1N1
A/Ohio/07/2009	A	H1N1
A/California/07/2009	A	H1N1
A/Solomon Islands/3/2006	A	H1N1
A/swine/Guangxi/13/2006	A	H1N2
A/California/04/2009	A	H1N1
A/California/04/2009	A	H1N1
A/Brisbane/59/2007	A	H1N1
A/Brevig Mission/1/1918	A	H1N1
A/Ohio/UR06-0091/2007	A	H1N1
A/mallard/Ohio/265/1987	A	H1N9
A/New York/1/1918	A	H1N1
A/New Caledonia/20/99	A	H1N1
A/WSN/1933	A	H1N1
A/duck/NZL/160/1976	A	H1N3
A/Beijing/262/1995	A	H1N1
A/USSR/90/1977	A	H1N1
A/Puerto Rico/8/34	A	H1N1

B

Influenza Strain/Control ID	Influenza Type	Subtype
A/Ohio/07/2009	A	H1N1
A/California/04/2009	A	H1N1
A/New York/18/2009	A	H1N1
A/California/06/2009	A	H1N1
A/Beijing/22808/2009	A	H1N1
A/Texas/05/2009	A	H1N1
A/England/195/2009	A	H1N1
A/mallard/Ohio/265/1987	A	H1N9
A/Brevig Mission/1/1918	A	H1N1
A/Ohio/UR06-0091/2007	A	H1N1
A/New York/1/1918	A	H1N1
A/USSR/90/1977	A	H1N1
A/swine/Guangxi/13/2006	A	H1N2
A/Egyptian goose/South Africa/AI1448/2007	A	H1N8
A/Texas/36/1991	A	H1N1
A/duck/NZL/160/1976	A	H1N3
A/New Caledonia/20/99	A	H1N1
A/Solomon Islands/3/2006	A	H1N1
A/Puerto Rico/8/34	A	H1N1
A/Brisbane/59/2007	A	H1N1
A/WSN/1933	A	H1N1

Figure SI-10. H1 HA0 and H1 HA1 variants printed on the protein microarray used for IgG, IgG1, and IgG2c antibody profiling. A) H1 HA0 descriptions: influenza strain, type, and subtype. **B)** H1 HA1 descriptions: influenza strain, type, and subtype. More details of each variant can be found in Supplementary Table 1.

Uploaded as a separate file:
Supplementary Table 1. Influenza protein microarray contents and raw data values from IgG, IgG1, and IgG2 probing. List of all protein microarray antigens including all tested HA HA0 and HA HA1 variants.

References

1. Lata, S., A. Reichel, R. Brock, R. Tampe, and J. Piehler, *High-affinity adaptors for switchable recognition of histidine-tagged proteins.* Journal of the American Chemical Society, 2005. **127**(29): p. 10205-10215.
2. Milder, F.J., M. Jongeneelen, T. Ritschel, P. Bouchier, I.J.M. Bisschop, M. de Man, D. Veldman, L. Le, B. Kaufmann, M.J.G. Bakkers, J. Juraszek, B. Brandenburg, and J.P.M. Langedijk, *Universal stabilization of the influenza hemagglutinin by structure-based redesign of the pH switch regions.* Proceedings of the National Academy of Sciences of the United States of America, 2022. **119**(6).
3. McMillan, C.L.D., S.T.M. Cheung, N. Modhiran, J. Barnes, A.A. Amarilla, H. Bielefeldt-Ohmann, L.Y.Y. Lee, K. Guilfoyle, G. van Amerongen, K. Stittelaar, V. Jakon, C. Lebas, P. Reading, K.R. Short, P.R. Young, D. Watterson, and K.J. Chappell, *Development of molecular clamp stabilized hemagglutinin vaccines for Influenza A viruses.* Npj Vaccines, 2021. **6**(1).
4. Maciola, A.K., M.A. Pietrzak, P. Kosson, M. Czarnocki-Cieciura, K. Smietanka, Z. Minta, and E. Kopera, *The Length of N-Glycans of Recombinant H5N1 Hemagglutinin Influences the Oligomerization and Immunogenicity of Vaccine Antigen.* Frontiers in Immunology, 2017. **8**.
5. Pietrzak, M., A. Maciola, K. Zdanowski, A.M. Protas-Klukowska, M. Olszewska, K. Smietanka, Z. Minta, B. Szewczyk, and E. Kopera, *An avian influenza H5N1 virus vaccine candidate based on the extracellular domain produced in yeast system as subviral particles protects chickens from lethal challenge.* Antiviral Research, 2016. **133**: p. 242-249.
